# Effects of smoking on the tissue regeneration-associated functions of human endometrial stem cells via a novel target gene SERPINB2

**DOI:** 10.1186/s13287-022-03061-1

**Published:** 2022-08-05

**Authors:** Se-Ra Park, Seong-Kwan Kim, Soo-Rim Kim, Wook-Joon Yu, Seung-Jin Lee, Hwa-Yong Lee

**Affiliations:** 1 Department of Health Sciences and Technology, GAIHST, Gachon University, Incheon, 21999 Republic of Korea; 2 Department of Molecular Medicine, School of Medicine, Gachon University, Incheon, 406-840 Republic of Korea; 3 Developmental and Reproductivoxicology Research Group, Korea Institute of Toxicology, Deajeon, 34114 Republic of Korea; 4 Division of Science Education, Kangwon National University, Chuncheon, 24341 Republic of Korea

**Keywords:** Smoking, Endometrium, Stem cells, Infertility, Self-renewal, Multilineage differentiation, Pluripotency, SERPINB2

## Abstract

**Background:**

Smokers directly inhale mainstream cigarette smoke, which contains numerous known and potential toxic substances, and thus, smoking is expected to have broad harmful effects that cause tissue injury and dysfunction. Interestingly, many studies have suggested that the recent decline in female fertility and increased rate of spontaneous abortion could be associated with increased smoking rates. Indeed, women that smoked for 10 years or more were reported to have a ~ 20% higher infertility rate than women that had never smoked. However, the reasons for the underlying harmful aspects of smoking on female fertility remain a matter of debate. Importantly, a previous study revealed that resident endometrial stem cell deficiency significantly limits the cyclic regeneration potential of endometrium, which, in turn, decreases successful pregnancy outcomes. In this context, we postulated that exposure to mainstream cigarette smoke extracts might decrease female fertility by inhibiting the functions of resident endometrial stem cells.

**Methods:**

We investigated whether cigarette mainstream smoke exposure directly inhibits various tissue regeneration-associated functions of endometrial stem cells, such as self-renewal, migration, pluripotency, and differentiation capacity in vitro. Next, we determined whether SERPINB2 mediates cigarette smoke-induced suppressive effects on various tissue regeneration-associated functions by depleting SERPINB2 expression with specific shRNA targeting SERPINB2. Mice were injected intraperitoneally with low (0.5 mg/kg) or high (1 mg/kg) doses of cigarette smoke extract (10 times for two weeks), and endometrial stem cells were then isolated from mice uterine tissues.

**Results:**

We found that exposure to cigarette smoke extracts remarkably suppressed various tissue regeneration-associated functions of endometrial stem cells, such as self-renewal, migration, multilineage differentiation ability, and pluripotency in vitro and in vivo by activating the SERPINB2 gene. Indeed, cigarette smoke-induced inhibitory effects on various endometrial stem cell functions were significantly abolished by SERPINB2 knockdown.

**Conclusions:**

These findings provide valuable information on the harmful effects of cigarette smoking on resident endometrial stem cells and hopefully will facilitate the developments of promising therapeutic strategies for subfertile or infertile women that smoke cigarettes.

**Supplementary Information:**

The online version contains supplementary material available at 10.1186/s13287-022-03061-1.

## Introduction

Cigarette smoke (CS) contains a complex mixture of around 7000 different chemicals, of which about 250 are pharmacologically active and quickly absorbed by tissues [1, 2]. Although epidemiological studies have consistently shown smoking has adverse effects on female reproductive processes [3–6], the mechanisms responsible are complicated by the physiological complexities of the human reproductive system. Close interactions between embryonic (placental/fetal), uterine endometrial (maternal), and ovarian (oocytes/endocrine) factors are essential for successful pregnancy outcomes. Furthermore, reports indicate cigarette smoking accelerates ovarian reserve depletion and leads to loss of reproductive function at an early age [7] and causes deleterious changes in the placenta [8–11] and fetus [12, 13] during pregnancy. Only a handful of studies have described the harmful effects of cigarette smoke on endometrial functions, and the effects of cigarette smoking on endometrial regenerative capacity have not been studied.

The endometrium is the innermost lining layer of the uterus and undergoes dynamic remodeling as it rapidly grows by up to ~ 7 mm per week during the menstrual cycle [14]. Successful embryo implantation and subsequent pregnancy in receptive uterine endometrium are largely dependent on the constant recruitment and activation of multipotent endometrial stem cells that can differentiate into multiple endometrial cell lineages [7, 15]. Importantly, a previous study revealed that cellular aging of resident endometrial stem cells decreases the cyclic regeneration potential of endometrium and subsequently reduces successful pregnancy rates [16]. Therefore, it is reasonable to hypothesize that cigarette smoke exposure might reduce endometrial regenerative capacity and adversely influence pregnancy outcomes by disrupting multiple cellular functions of endometrial stem cells. However, little information is available on the suppressive effects of cigarette smoke exposure on resident stem cells, though it has been established that exposure to whole cigarette smoke [17, 18] and even its individual components (including nicotine) [19–21] remarkably inhibits some cellular functions of various stem cell types. Furthermore, no evidence has been presented that cigarette smoke directly affects the tissue regeneration-associated functions of endometrial stem cells, and thus, it is difficult to conclude whether these inhibitions of stem cell functions are attributable to the direct suppressive effects of cigarette smoke or smoking-induced secondary (indirect) outcomes, such as chronically elevated levels of pro-inflammatory factors [22, 23] and/or reactive oxygen species (ROS) [24, 25].

Consistent with this hypothesis, we found that cigarette smoke exposure significantly suppresses various tissue regeneration-associated functions of endometrial stem cells, such as growth potential, cellular aging, multilineage differentiation, migration capacities, and pluripotency in vitro and in vivo. We also investigated the mechanism underlying these smoking-induced suppressive effects on endometrial stem cell functions. Importantly, cigarette smoke exposure significantly increased expressions of SERPINB2, which have recently been demonstrated to be associated with the regulations of various stem cell functions [26, 27]. In the present study, smoking-induced inhibitions of tissue regeneration-associated functions were significantly abolished by SERPINB2 knockdown, suggesting that SERPINB2 may act as a key regulator of the harmful effects of smoking on various endometrial stem cell functions. Taken together, these findings provide valuable information regarding the harmful effects of smoking on tissue resident endometrial stem cells and delineate novel mechanisms activated in response to cigarette smoking. It is hoped that this study will aid the development of therapeutic strategies that prevent or treat cigarette smoking-related infertility.

## Materials and methods

### Isolation and culture of human endometrial stem cells from uterine tissues

Human endometrial stem cells were obtained from endometrial tissues of uterine fibroid patients with written informed consent from the patients and approval of the Gachon University Institutional Review Board (IRB No: GAIRB2018-134). To isolate human endometrial stem cells, endometrial tissue from women undergoing hysterectomy for treatment of uterine fibroids was minced into small pieces, and then the small pieces were digested in DMEM containing 10% FBS and 250 U/ml type I collagenase for 5 h at 37 °C in a rotating shaker. The digestion mixture was then filtered through a 40-µm cell strainer to separate spindle-shaped endometrial stem cells from epithelial gland fragments and undigested tissue. Among endometrial cells that have passed through a 40-µm cell strainer, endometrial stromal cells can be removed by quickly attaching (within 30 min) to cell culture dish. Unattached endometrial stem cells were harvested and then cultured in EBM-2 medium (Lonza) with EGM-2 supplements at 37 °C and 5% CO_2_ [28]. In this study, we used endometrial stem cells under passage 15.

### Preparation of aqueous cigarette smoke extract

Standard cigarette used in this study was provided from the Kentucky Tobacco Research and Development Center (University of Kentucky, Lexington, NY). The cigarette extracts production method was described by Carp and Janoff with some modification [ref 14]. The cigarette smoke extracts (10%) were produced by bubbling cigarette smoke with one cigarette/minute in 10 ml of culture media as described previously [29, 30]. The extraction pH was adjusted to pH 7.4 and filtered with 0.45-μm filter (25 mm Acrodisc; Pall Corporation, Ann Arbor, MI). The cigarette smoke extracts were normalized with OD 0.74 ± 0.05 at 320 nm wavelength. The absorbance pattern at λ320 showed little variation between the batches. The extracts were prepared freshly for experiments and diluted with culture media immediately before use. Vehicle control medium was prepared with 10 ml of culture media bubbling air, adjusting the pH to 7.4, and filtered in 0.45-μm filter.

### Cell proliferation assay

The MTT assay was used to determine the anti-proliferative capacity of cigarette smoke exposure. Briefly, cells (1 × 10^4^ cells/well) were seeded in 48-well plates in EBM-2 medium (Lonza) with EGM-2 supplements. After 24 h of incubation, the plates were washed with PBS, then the cells were treated with cigarette smoke extract or vehicle for 72 h in serum and supplement-free EBM-2 medium. Thereafter, 50 µl of MTT solution (Sigma-Aldrich, M5655, 5 mg/ml in PBS) was added into each well and cells were incubated at 37℃ for another 3 h. The viable cells were measured at 570 nm using a VersaMax Microplate Reader.

### Senescence-associated beta-galactosidase (SA β-gal) staining

SA β-gal staining was performed as previously described [31]. The endometrial stem cells were seeded on 6-well plates at a density of 1 × 10^5^ cells/well. The cells were incubated for 3 days until they reached the appropriate confluency. The cells were then washed twice with PBS and fixed with 0.5% glutaraldehyde in PBS for 5 min. The cells were then washed with PBS containing 1 mM MgCl_2_ and stained with *X*-gal solution [1 mg/ml *X*-gal, 0.12 mM K_3_Fe(CN)_6_, 1 mM MgCl_2_ in PBS, pH 6.0] overnight at 37 °C.

### In vitro cell migration assay

Cells were plated at 1 × 10^5^ cells/well in 200 μL of culture medium in the upper chambers of transwell permeable supports (Corning Inc., Corning, NY, USA) to track the migration of cells. The transwell chambers had 8.0-μm pores in 6.5-mm-diameter polycarbonate membranes and used a 24-well plate format. Non-invading cells on the upper surface of each membrane were removed by scrubbing with laboratory paper. Migrated cells on the lower surface of each membrane were fixed with 4% paraformaldehyde for 5 min and stained with hematoxylin for 15 min. Later, the number of migrated cells was counted in three randomly selected fields of the wells under a light microscope at 50X magnification. To calculate the chemotactic index, the number of cells that migrated in response to the treatment of cigarette smoke extract was divided by the number of spontaneously migrating cells.

### Real-time PCR

Total RNA was extracted using TRIzol reagent (Invitrogen) according to the manufacturer’s protocol. RNA purity was verified by measuring the 260/280 absorbance ratio. The first-strand cDNA was synthesized with 1 μg of total RNA using SuperScript II (Invitrogen), and one-tenth of the cDNA was used for each PCR mixture containing Express SYBR-Green qPCR SuperMix (BioPrince, Seoul, South Korea). Real-time PCR was performed using a Rotor-Gene Q (Qiagen). The reaction was subjected to 40-cycle amplification at 95 °C for 20 s, 60 °C for 20 s and 72 °C for 25 s. The relative mRNA expression of the selected genes was normalized to that of PPIA and quantified using the ΔΔCT method. The sequences of the PCR primers are listed in Table 1.

**Table 1:** Primer sequences for quantitative RT-PCR

Gene	Gene bank no.	Direction	Primer sequence
Human PPIA	NM_021130	F	TGCCATCGCCAAGGAGTAG
		R	TGCACAGACGGTCACTCAAA
Human IL6	NM_000600	F	GGTACATCCTCGACGGCATCT
		R	GTGCCTCTTTGCTGCTTTCAC
Human P16	NM_000077	F	CTACTGAGGAGCCAGCGTCT
		R	CTGCCCATCATCATGACCT
Human P18	NM_001262	F	TGGGTCTTCCGCAAGAACTC
		R	TGGCAGCCAAGTGCAAGGGC
Human P21	NM_000389	F	ACAGCAGAGGAAGACCATGTGGACC
		R	CGTTTTCGACCCTGAGAGTCTCCAG
Human C-MYC	NM_002467	F	AAAGGCCCCCAAGGTAGTTA
		R	GCACAAGAGTTCCGTAGCTG
Human KLF4	NM_001314052	F	GAACTGACCAGGCACTACCG
		R	TTCTGGCAGTGTGGGTCATA
Human NANOG	NM_024865	F	TGGGATTTACAGGCGTGAGC
		R	AAGCAAAGCCTCCCAATCCC
Human OCT4	NM_002701	F	AGCCCTCATTTCACCAGGCC
		R	TGGGACTCCTCCGGGTTTTG
Human SOX2	NM_003106	F	AAATGGGAGGGGTGCAAAAGAGGAG
		R	CAGCTGTCATTTGCTGTGGGTGATG
Human SERPINB2	NM_001143818	F	ACCCCCATGACTCCAGAGAACT
		R	GAGAGCGGAAGGATGAATGGAT
Mouse HPRT	NM_013556	F	GCCTAAGATGAGCGCAAGTTG
		R	TACTAGGCAGATGGCCACAGG
Mouse C-MYC	NM_010849	F	CGCACACACAACGTCTTGGA
		R	AGGATGTAGGCGGTGGCTTT
Mouse KLF4	NM_010637	F	GGTGCAGCTTGCAGCAGTAA
		R	AAAGTCTAGGTCCAGGAGGT
Mouse NANOG	NM_028016	F	GCCTTACGTACAGTTGCAGC
		R	TCACCTGGTGGAGTCACAGA
Mouse OCT4	NM_013633	F	GCATTCAAACTGAGGCACCA
		R	AGCTTCTTTCCCCATCCCA
Mouse SOX2	NM_011443	F	GAAGCGTGTACTTATCCTTCTTCAT
		R	GAGTGGAAACTTTTGTCCGAGA

### Protein isolation and western blot analysis

**Fig. 1:**
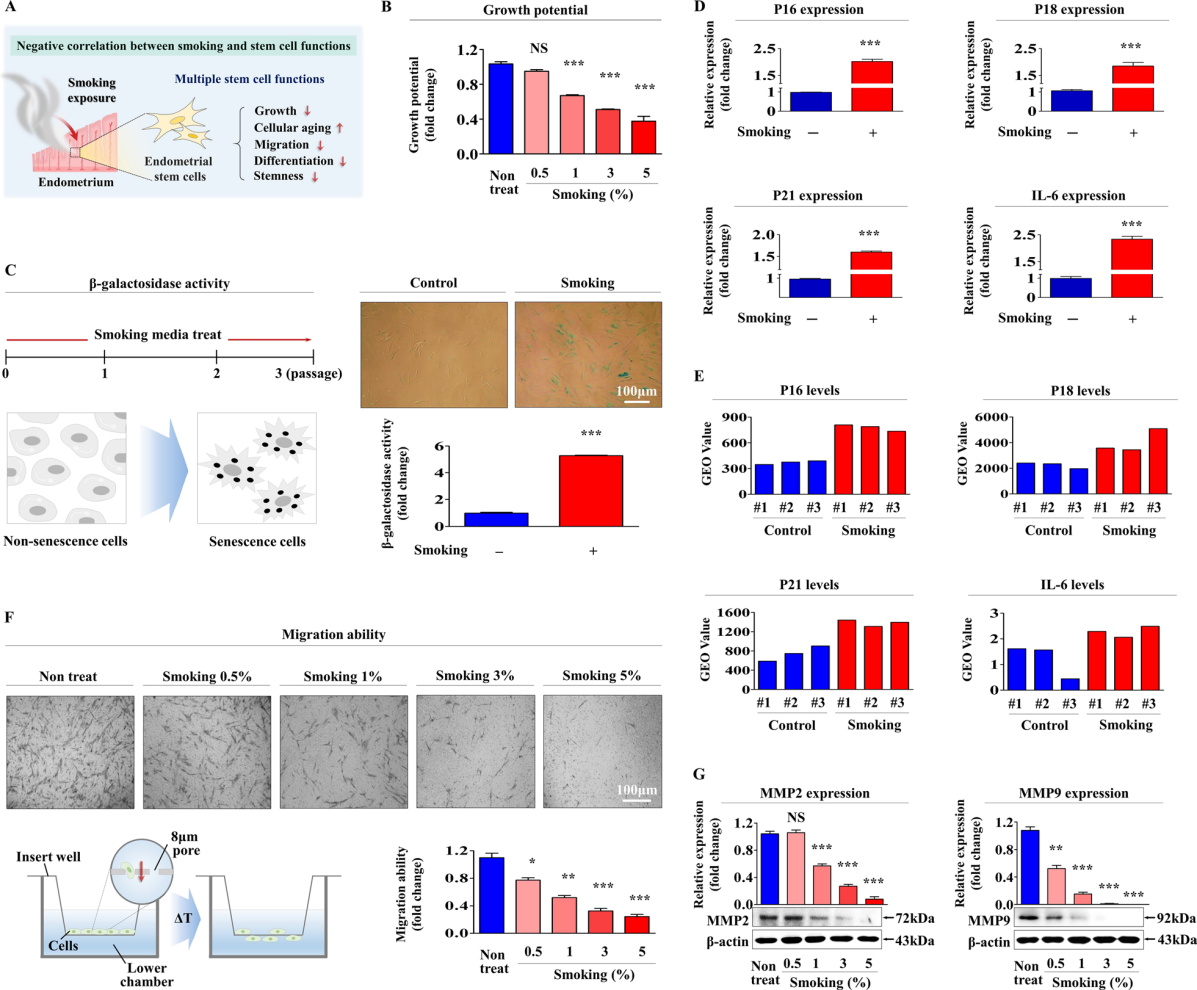
Cigarette smoke exposure significantly reduced the proliferations and migrations of endometrial stem cells and induced cellular senescence. We postulated that cigarette smoke exposure suppresses various tissue regeneration-associated functions of endometrial stem cells, such as proliferation, senescence, migration potential, and differentiation capacity, and pluripotency/stemness (**A**). The inhibitory effects of cigarette smoke extract (0.5, 1, 3, 5%) on the self-renewal capacity of endometrial stem cells were analyzed using a 72-h MTT assay. Proliferative potentials (%) were calculated by expressing numbers of viable cells after treatment with cigarette smoke extract (0.5, 1, 3, 5%) as percentages of numbers after treatment with vehicle (**B**). Effects of cigarette smoke exposure on the senescence (aging) of endometrial stem cells were assessed by analyzing senescence-associated β-galactosidase (SA-β-Gal) activity in cells consecutively exposed to 1% cigarette smoke extract treatment for 72 h (**C**). Effects of cigarette smoke exposure on the mRNA levels of various senescence (aging) markers (p16, p18, p21, and IL-6) were determined by real-time PCR (**D**). The GEO (Gene Expression Omnibus) metadata respiratory (https://www.ncbi. nlm.nih.gov/geo/) was analyzed to assess relationships between cigarette smoking and levels of various senescence (aging) markers (**E**). Endometrial stem cells were exposed to 1% cigarette smoke extract for 72 h, and inhibitory effects on migration were determined using transwell assays (**F**). The relative protein levels of key regulators of cell migration (MMP-2/9) after exposure or not to cigarette smoke exposure were analyzed by western blotting (**G**). β-actin was used as the internal control for western blotting, and PPIA was used as the housekeeping gene real-time PCR. All experiments were performed in triplicate. Data are presented as means ± standard deviations (SDs). *, *p* < 0.05; **, *p* < 0.005; and ***, *p* < 0.001 (two-sample *t* test)

The protein expression levels were determined by western blot analysis as previously described [32]. Cells were lysed in a buffer containing 50 mM Tris, 5 mM EDTA, 150 mM NaCl, 1 mM DTT, 0.01% NP 40, and 0.2 mM PMSF. The protein concentrations of the total cell lysates were measured by using bovine serum albumin as a standard. Samples containing equal amounts of protein were separated via sodium dodecyl sulfate–polyacrylamide gel electrophoresis (SDS-PAGE) and then transferred onto nitrocellulose membranes (Bio-Rad Laboratories). The membranes were blocked with 5% skim milk in Tris-buffered saline containing Tween-20 at RT. Then, the membranes were incubated with primary antibodies against β-actin (Abcam, MA, USA, ab189073), MMP-2 (Cell signaling #4022), MMP-9 (Cell Signaling #13,667), SERPINB2 (Abcam, MA, USA, ab47742), and caspase-3 (Cell signaling, MA, USA, #9662), overnight at 4 °C and then with HRP-conjugated goat anti-rabbit IgG (BD Pharmingen, San Diego, CA, USA, 554,021) and goat anti-mouse IgG (BD Pharmingen, 554,002) secondary antibodies for 60 min at RT. Antibody-bound proteins were detected using ECL reagents. The human endometrial stem cells were used in Figs. 1, 4, 5 and 6. The mouse endometrial stem cells were used in Fig. 8. The mouse adipose stem cells are used in Additional file 1: Fig. S7. The mouse bone marrow stem cells are used in Additional file 1: Fig. S8.

### Immunofluorescent staining

Samples were fixed with 4% paraformaldehyde for fluorescent staining. Samples were permeabilized with 0.4 M glycine and 0.3% Triton X-100, and nonspecific binding was blocked with 2% normal swine serum (DAKO, Glostrup, Denmark). Staining was performed as described previously [33], using the primary anti-Phalloidin (Cytoskeleton Inc.) antibody. Samples were examined by fluorescence microscopy (Zeiss LSM 510 Meta). The calculation of expression was based on red fluorescence areas and density divided by cell number, as determined from the number of DAPI-stained nuclei, in three randomly selected fields for each sample from a total of three independent experiments.

### Osteogenic differentiation

Endometrial stem cells were incubated in DMEM high-glucose medium supplemented with 0.1 µM dexamethasone, 10 mM β-glycerophosphate, 50 µM ascorbate, and 10% FBS with or without cigarette smoke extract treatment. Endometrial stem cells were grown for 3 weeks, with medium replacement twice a week. Differentiated cells were stained with Alizarin Red S to detect de novo formation of bone matrix. Alizarin red S in samples was quantified by measuring the optical density (OD) of the solution at 570 nm.

### Adipogenic differentiation

Endometrial stem cells were incubated in DMEM low-glucose medium supplemented with 500 µM methylxanthine, 5 µg/mL insulin, and 10% FBS with or without cigarette smoke extract treatment. Endometrial stem cells were grown for 3 weeks, with medium replacement twice a week. Lipid droplet formation was confirmed by Oil Red O staining. Relative quantification of lipid droplet formation was determined by absorbance measurement at 500 nm.

### Mitochondrial respiration and glycolytic capacity analysis

Using the Seahorse XF analyzer (Seahorse Bioscience, North Billerica, MA), in real time, mitochondrial oxidative phosphorylation and glycolytic flux can be analyzed by measuring OCR and ECAR of cells as they respond to substrates and metabolic inhibiting agents according to manufacturer’s instructions [34]. ATP synthase inhibitor oligomycin (a complex V blocker) is added to inhibit coupled respiration. FCCP (a mitochondrial uncoupler) is added to collapse mitochondrial membrane potential (Δψm). Rotenone (an inhibitor of complex I of the electron transport chain) and antimycin A (an inhibitor of complex III of the electron transport chain) were add to block mitochondrial respiration completely. To analyze real-time glycolytic rates, Seahorse XF glycolytic rate assay utilizes both (extracellular acidification rate) ECAR and OCR measurements to evaluate the glycolytic proton efflux rate (glycoPER) of the cells, in which the cells were incubated in glucose-free media followed by the addition of rotenone, antimycin A, and finally 2-deoxyglucose (2-DG, glycolysis inhibitor). OCR and ECAR were described as absolute rates (pmoles/min for OCR and mpH/min for ECAR) and normalized against cell counts as a percentage of the baseline oxygen consumption.

### SERPINB2 knockdown

Small hairpin RNA (shRNA: accession No. NM_002575) targeting SERPINB2 and scrambled shRNA (shCon) were purchased from Bioneer (Daejeon, South Korea). For efficient SERPINB2 transfection, reverse transfection was performed using Lipofectamine 2000 (Invitrogen) according to the manufacturer’s protocol. Briefly, shRNA targeting SERPINB2 (3 μg/ml) was mixed with 3 μl transfection reagent Lipofectamine 2000 in Gibco opti-MEM media without FBS and antibiotics. 5 h after transfection, opti-MEM was replaced with fresh EGM-2 medium with 10% FBS. We chose the SERPINB2 shRNA that is most effective in mRNA levels from three shRNA designed from the target sequence and determined by qRT-PCR analysis.

### Flow cytometry

FACS analysis and cell sorting were performed using FACSCalibur and FACS Aria machines (Becton Dickinson, Palo Alto, CA), respectively. FACS data were analyzed using FlowJo software (Tree Star, Ashland, OR). Antibodies to the following proteins were used: PE-conjugated CD34 (MACS; Miltenyi Biotec, 30-081-002), CD44 (MACS; Miltenyi Biotec, 130-095-180), CD45 (MACS; Miltenyi Biotec, 130-080-201), CD73 (MACS; Miltenyi Biotec, 130-095-182), CD105 (MACS; Miltenyi Biotec, 130-094-941), CD140b (MACS; Miltenyi Biotec, 130-105-279), and W5C5 (MACS; Miltenyi Biotec, 130-111-641). The FACS gates were established by staining with an isotype antibody or secondary antibody.

### Growth factor antibody array

The assay was performed following the manufacturer's protocol (Abnova AA0089). Briefly, cigarette smoke extract or vehicle-treated protein samples were incubated with antibody membranes overnight at 4 °C. After washing 3 times with wash buffer, the membranes were incubated with biotin-conjugated anti-cytokine antibodies overnight at 4 °C. The membranes were then washed 3 times and incubated with HRP-conjugated streptavidin. Chemiluminescence was used to detect signals of the growth factors spotted on the nitrocellulose membrane.

### Analysis of gene expression omnibus (GEO) database

Gene Expression Omnibus (GEO) (https://www.ncbi.nlm.nih.gov/geo/) is a freely distributed database repository of high-throughput gene expression data generated by genome hybridization arrays, chip sequencing, and DNA microarrays [35, 36]. Researchers provide their experimental results in four categories: experimental designs, sample, platform, and raw data. Clinical or experimental samples within an each dataset are further organized based on various experimental subgroups, such as treatment, physiologic condition, and disease state. These categorized biological data are presented as a “GEO profile,” which includes the dataset title, gene annotation, a chart depicting the expression levels, and rank for that gene across each sample [37]. The expression profiles of AR, EGF, FGF4/7, HGF, IGFBP4/6, NT-4, PDGFR β, TGF-β3, and VEGFR3 in response to various toxic exposures were analyzed according to previously established procedures [37].

### Ingenuity pathway analysis

The analysis for SERPINB2-related genes was performed with Ingenuity Pathway Analysis (IPA) version 2.0 software (Ingenuity Systems, Redwood City, CA). Differentially expressed genes (*t* test, *P* < 0.005) between toxicant-treated cells and non-treated cells were subjected to SERPINB2-related genes analysis (GSE69851). The significance of each molecule was measured by Fisher's exact test (*P* value), which was used to identify differentially expressed genes from the microarray data that overlapped with genes known to be regulated by a molecule. The activation score (*z s*core) was used to show the status of predicted molecules by comparing the observed differential regulation of genes (“up” or “down”) in the microarray data relative to the literature-derived regulation direction, which can be either activating or inhibiting.

### GeneMANIA algorithm-based bioinformatics analysis

To further analyze eleven genes that interact with or directly regulate self-renewal, pluripotency, and migratory capacity, we imported all identified genes and their corresponding accession numbers into GeneMANIA (http://www.genemania.org). To find gene interactions, we considered several factors including co-expression, colocalization, and genetic interactions. From this list, we selected the genes AR, EGF, FGF4/7, HGF, IGFBP4/6, NT-4, PDGFR β, TGF-β3, and VEGFR3 to test their involvement in regulating various cellular functions, such as elf-renewal, pluripotency, and migratory capacity.

### Evaluation of the effects of cigarette smoke exposure in animal model

All of the animal experiments were approved and conducted in accordance with the Institutional Animal Care and Use Committee (KCDC-029-20-2A) of Korea Centers for Disease Control and Prevention. The mice were randomly divided into control (vehicle) and cigarette smoke extract low (0.5 mg/kg) and high (1 mg/kg) treatment groups. ICR mice were exposed to cigarette smoke extract or vehicle (PBS) through intraperitoneal injection two times. The mice were anesthetized and exsanguinated by cardiac puncture, and then endometrial stem cells were isolated from uterine endometrium. In this study, an in vivo experiment was conducted using 8-week-old ICR mice in each group.

### Statistical analysis

All the statistical data were analyzed in GraphPad Prism 5.0 (GraphPad Software, San Diego, CA) and evaluated using two-tailed Student’s *t* tests. Values of *P* < 0.05 were considered to indicate statistical significance.

## Results

### Cigarette mainstream (directly inhaled) smoke exposure remarkably inhibits the tissue regeneration-associated functions of human endometrial stem cells in vitro

**Fig. 2:**
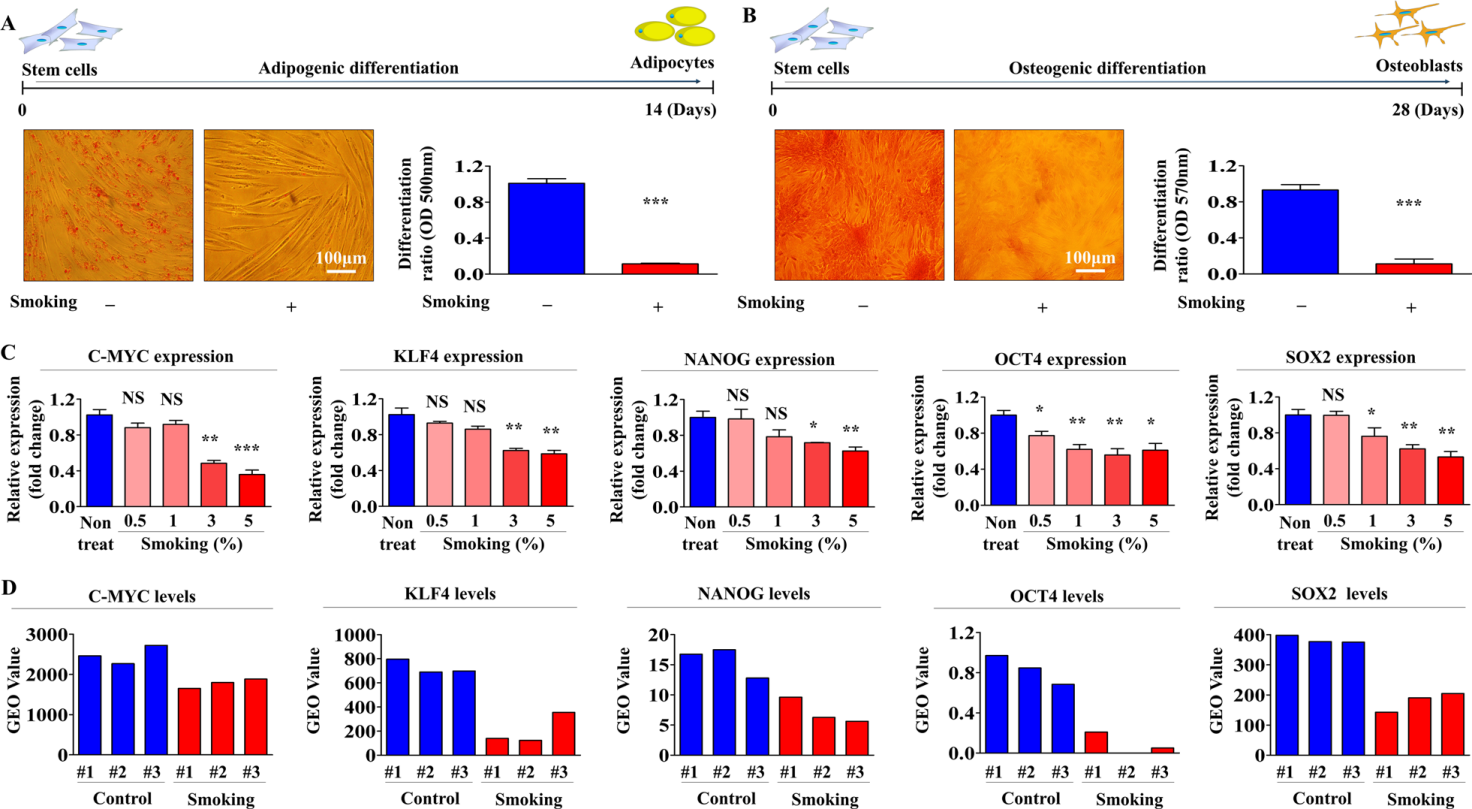
Cigarette smoke exposure significantly reduced differentiation capacity and the pluripotency/stemness of endometrial stem cells. Human endometrial stem cells were pretreated with or without 1% cigarette smoke extract and then cultured in adipocyte or osteoblast differentiation medium. The suppressive effects of cigarette smoke exposure on adipocyte (**A**) and osteoblast (**B**) differentiation were analyzed by oil red O and alizarin red staining, respectively. The relative quantification of calcium mineral contents and lipid droplet formation within differentiated cells was determined by measuring absorbance at 500 nm and 570 nm, respectively. The suppressive effects of cigarette smoke exposure on the mRNA levels of various pluripotency-associated genes (C-MYC, KLF4, NANOG, OCT4, and SOX2) were analyzed by real-time PCR (**C**). The GEO metadata respiratory was analyzed to investigate the relationships between cigarette smoke exposure and levels of various pluripotency-associated genes (**D**). PPIA was used as the housekeeping gene for real-time PCR. All experiments were performed in triplicate, and data are presented as means ± SDs. *, *p* < 0.05; **, *p* < 0.005; and ***, *p* < 0.001 (two-sample *t* test)

To assess the inhibitory effects of cigarette smoke exposure, we isolated endometrial stem cells from human uterine tissue (Additional file 1: Fig. S1A) and then investigated their biological characteristics by analyzing multiple negative and positive lineage-specific surface antigens. Four positive surface antigens (CD44, CD73, CD105, and CD140b) were predominantly expressed (on > 99% of cells), and two lineage-specific antigen (CD146 and susD2) positive subpopulations were detected on 76–79% of cells (Additional file 1: Fig. S1B), which suggested that stem cells in human uterine tissues are a mixture of at least two cell types. Their multilineage differentiation abilities were confirmed by inducing adipogenic (Additional file 1: Fig. S1C) and osteogenic (Additional file 1: Fig. S1D) differentiation, which suggests the endometrial stem cells used possessed transdifferentiation ability of multipotent stem cells. Next, we investigated whether cigarette mainstream smoke exposure directly inhibits various tissue regeneration-associated functions of endometrial stem cells in vitro (Fig. 1A). Because cigarette smoke contains many different chemicals, cigarette smoke aqueous aerosol extract is commonly used to assess smoking-induced in vitro toxicity [24, 38, 39]. Cigarette smoke extract was generated by bubbling filtered mainstream smoke from cigarettes into culture media [40]. Importantly, the self-renewal capacity of endometrial stem cells was significantly and dose-dependently reduced by cigarette smoke exposure (Fig. 1B). The most widely used biomarker for the measurement of cellular senescence (aging) in vitro is the increased senescence-associated β-galactosidase (SA-β-Gal) activity [41]. Therefore, to investigate whether cigarette smoke exposure can accelerate cellular senescence (aging), endometrial stem cells were continuously passaged with or without cigarette mainstream smoke exposure and then analyzed SA-β-Gal activity (Fig. 1C). Besides the elevated activity of SA-β-Gal, increased levels of cytokines or cell cycle regulators, such as IL-6, p16^INK4A^, p18, and p21^CIP1^, are also commonly accepted biomarkers of cellular senescence or aging [42]. The mRNA levels of the senescence-associated cytoplasmic proteins were significantly increased by cigarette smoke exposure (Fig. 1D). We also analyzed metadata in the gene expression omnibus (GEO; an international public repository) for relationships between cigarette smoke exposure and levels of these senescence-associated genes, and found the expressions of these typical aging markers are markedly enhanced by cigarette smoking (Fig. 1E). Interestingly, transwell assays revealed cigarette smoke exposure inhibited the migration of endometrial stem cells (Fig. 1F). To further investigate the inhibitory effect of cigarette smoke on the migration potential of endometrial stem cells, western blotting was conducted to assess the expressions of MMP-2 and MMP-9 (matrix metalloproteinase-2 and matrix metalloproteinase-9), which play key regulatory roles in cell migration and invasion (Fig. 1G). Cigarette smoke exposure was found to remarkably suppress the ability of endometrial cells to differentiate into adipocytes (Fig. 2A) and osteoblasts (Fig. 2B). Additionally, the mRNA levels of various pluripotency/stemness-related genes, such as C-MYC, KLF4, NANOG, OCT4, and SOX2, were significantly and dose-dependently reduced by cigarette smoke exposure (Fig. 2C), which concurred with GEO repository data (Fig. 2D). These results indicate that cigarette smoking suppresses various tissue regeneration-associated functions, including self-renewal, multilineage differentiation potential, migration, and pluripotency, of endometrial stem cells.

### Endometrial stem cell metabolic activities (energy production) are significantly accelerated by cigarette smoke exposure

**Fig. 3:**
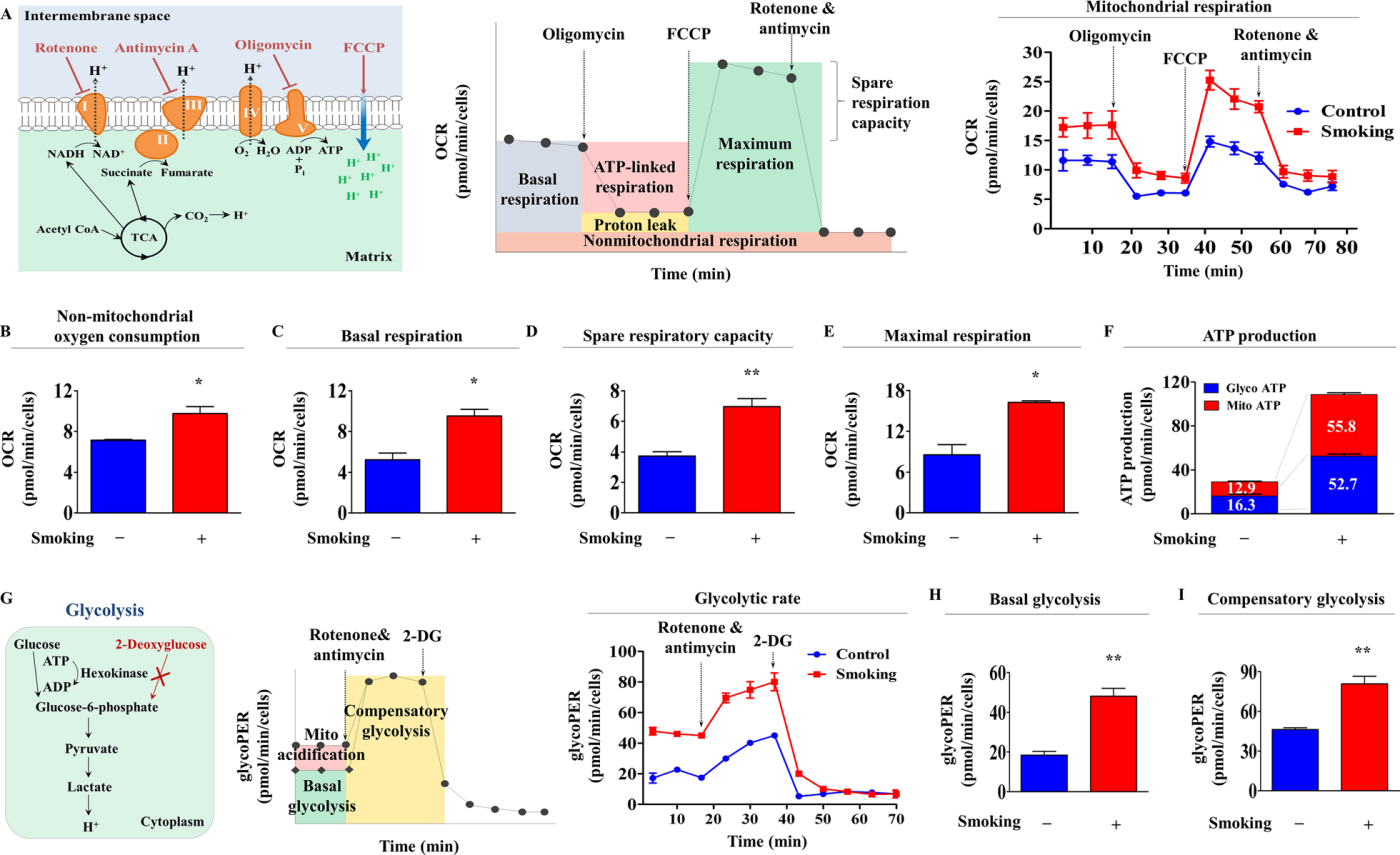
Cigarette smoke exposure significantly increased metabolic activity (mitochondrial oxidative phosphorylation and glycolysis) in endometrial stem cells. To investigate the effect of cigarette smoke exposure on metabolic activity (energetic status) in endometrial stem cells, mitochondrial oxidative phosphorylation and glycolysis were measured after exposing or not exposing cells to cigarette smoke extract. Oxidative phosphorylation patterns of endometrial stem cells exposed or not to 1% cigarette smoke extract for 72 h were analyzed by measuring twelve consecutive oxygen consumption rates (OCR) using an XF analyzer (Seahorse Bioscience). A schematic of real-time analysis of mitochondrial oxidative phosphorylation using a Seahorse XF analyzer (**A**). Endometrial stem cells were seeded in multiplates at a density of 2 × 10.^4^ cells per well, incubated overnight in complete growth medium containing 10% FBS, and gently washed 3 times with seahorse XF medium. Cells were then treated with ATP synthase blocker oligomycin (a complex V inhibitor, 1.5 μM) to prevent coupled respiration (respiration linked to ATP production), FCCP (a potent uncoupler of mitochondrial oxidative phosphorylation, 2 μM) to reduce the electrochemical proton gradient (Δψm), rotenone (a complex I blocker of the electron transport chain, 0.5 uM), and antimycin A (a complex III blocker of the electron transport chain, 0.5 uM) to completely prevent mitochondrial oxidative phosphorylation. These blockers were added automatically into the multiplate, and OCR was analyzed every 15 min. Overall mitochondrial respiratory responses of endometrial stem cells were significantly elevated by cigarette smoke exposure and showed increased levels of nonmitochondrial oxygen consumption (**B**). Cigarette smoke exposure markedly enhanced the basal respiration rate (**C**), reserve respiratory capacity (**D**), and maximal respiratory potential (**E**). ATP synthesis was markedly increased by cigarette smoke exposure in mitochondria and cytosol (**F**). A schematic of cytosolic glycolysis analysis in real time using a Seahorse XF analyzer (**G**). The Seahorse XF glycolytic rate assay was used to measure OCR and ECAR (extracellular acidification rate) in real-time to determine glycolytic proton efflux rates (glycoPER) of endometrial stem cells, which were cultured in glucose-free medium after treatment with antimycin A (1.67 μM), rotenone, and 50 mM 2-deoxyglucose (2-DG, glycolysis blocker). These agents were added automatically to multiplates, and ECAR was analyzed at 15 min intervals. The overall percentage (%) of PER from glycolytic rate indicates the contribution made by glycolysis to total ECAR (**G**). Compensatory glycolysis is the glycolytic rate in cells after blocking oxidative phosphorylation and supporting compensatory energy synthesis through glycolysis to meet energy requirements. Cigarette smoke exposure also markedly increased basal glycolysis (**H**) and compensatory glycolysis (**I**). ECAR values were normalized with respect to cell counts in each well. Bar graphs represent the averages of three independent experiments. Significant differences are presented as *p < 0.05, **p < 0.005, and ***p < 0.001 (two-sample t test)

Metabolic activities (energy status) are involved in various stem cell functions, including self-renewal, pluripotency/stemness, and multilineage differentiation capacity, by regulating energy (ATP) synthesis through cytosolic glycolysis and/or mitochondrial oxidative phosphorylation [43–45]. In addition, exposure to various environmental factors can alter the metabolic activities (energy status) of many cell types [46, 47]. Mitochondrial oxidative phosphorylation capacity is a key indicator of overall cellular health and energy (ATP)-producing potential [48], and thus, to investigate the effect of cigarette smoke exposure on the cellular energy phenotype of endometrial stem cells, we performed kinetic analysis of mitochondrial respiration using a Seahorse XF analyzer (Agilent), which provides quantitative real-time measurements of the oxygen consumption rates (OCR) of live cells [49]. The ATP synthase inhibitor oligomycin (also known as a complex *V* specific inhibitor) was used to block coupled respiration (or routine respiration, as referred to by Pesta et al. [24]) in mitochondria, and FCCP (an uncoupler of mitochondrial oxidative phosphorylation) was used to eliminate mitochondrial membrane potentials (Δψm), which cause direct proton transport across the inner mitochondrial membrane. FCCP dramatically increases oxygen consumption without ATP synthesis [48] and enables maximal mitochondrial respiration capacity to be assessed. In endometrial stem cells, cigarette smoke exposure markedly enhanced mitochondrial respiratory capacity (Fig. 3A), increased nonmitochondrial oxygen consumption (Fig. 3B), and also significantly increased basal respiration (Fig. 3C), spare respiratory capacity (Fig. 3D), and maximal respiration (Fig. 3E), which are used to assess reserve ATP synthesis potential produced by mitochondrial oxidative phosphorylation to adapt sudden energy (ATP) demands [50]. Furthermore, overall ATP synthesis was significantly and consistently increased by cigarette smoke exposure (Fig. 3F). Because anaerobic glycolysis of glucose to pyruvate by cells produces protons and lactate [51, 52], we analyzed the glycolytic activity of endometrial stem cells exposed or not to cigarette smoke by measuring real-time extracellular acidification rates (ECARs) after treating cells with 2-deoxyglucose (2-DG) to inhibit glycolytic activity, and thus, obtain information on basal ECAR. [53] In addition, complex I and III of the mitochondrial electron transport chain were blocked with rotenone and antimycin A, respectively, to completely inhibit mitochondrial respiration and oxygen consumption [54]. A schematic summary of real-time glycolytic rates obtained using a Seahorse XF analyzer is provided in Fig. 3G. Real-time measurements of glycolytic activity revealed that cigarette smoke-exposed endometrial stem cells had greater glycolytic activity than nonexposed cells (Fig. 3G). Cigarette smoke exposure also significantly elevated basal glycolytic rate (Fig. 3H) and compensatory glycolytic capacity (Fig. 3I). These results suggest that cigarette smoking might increase aerobic (oxidative phosphorylation) and anaerobic (glycolysis) energy production, and in turn, accelerate the cellular aging (senescence) of stem cells [55–57].

### SERPINB2 and its associated signaling networks are aberrantly activated by cigarette smoke exposure in endometrial stem cells

**Fig. 4:**
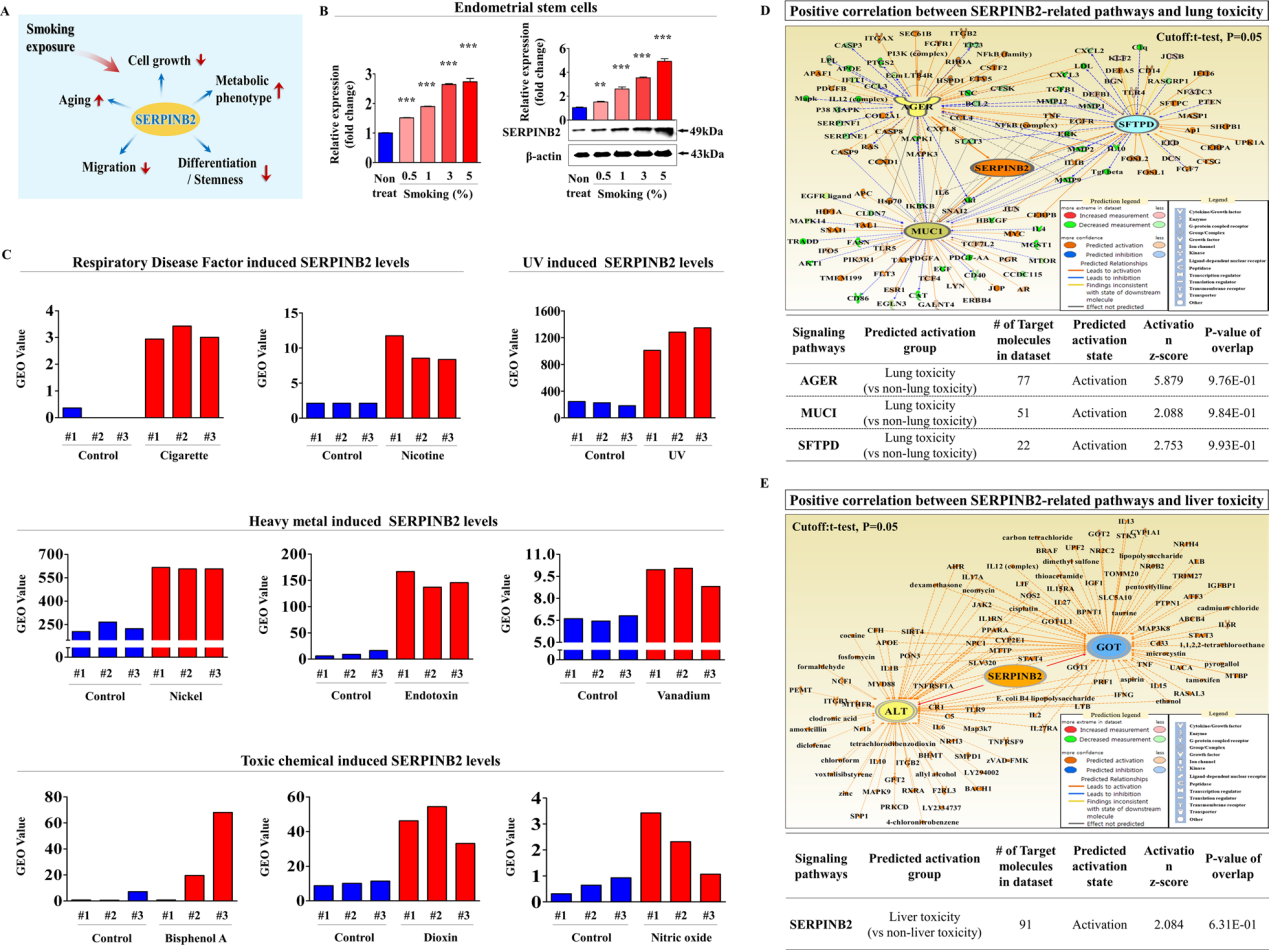
Cigarette smoke exposure promoted SERPINB2 expression levels in endometrial stem cells. We postulated that SERPINB2 functions as a key regulator of smoking-induced toxic response (**A**). Cells were treated with cigarette smoke extract at 0.5, 1, 3, or 5% for 72 h. Real-time PCR and western blotting were conducted to investigate the effects of cigarette smoke exposure on the mRNA and protein levels of SERPINB2 (**B**). GEO respiratory metadata were analyzed to investigate interactions between elevated SERPINB2 expression and exposures to toxic agents, such as radiation, heavy metals, air pollution, and toxic substances (**C**). Differentially activated genes in toxicant exposed lung cells and nonexposed lung cells were analyzed using IPA software to investigate the activation statuses (intermediate, inactive, or activate) of SERPINB2 (GSE62564)-associated signaling molecules/transcription factors (**D**). Differentially activated genes in toxicant exposed liver cells and nonexposed liver cells were analyzed using IPA software to investigate the activation status of SERPINB2 (GSE62564)-associated signaling molecules/transcription factors (**E**). β-actin was used as the internal control, and PPIA as the housekeeping gene for real-time PCR. All experiments were performed in triplicate. Data are presented as means ± SDs. *, *p* < 0.05; **, *p* < 0.005; and ***, *p* < 0.001 (two-sample *t* test)

We previously reported that SERPINB2 (*a* ~ 60 kDa serine protease inhibitor) might serve as a reliable marker of toxic response to various hazardous compounds in multiple types of cancer stem cells [58] and cord blood stem cells [27]. Therefore, to determine whether SERPINB2 functions as a key regulator of smoking-induced toxic response (Fig. 4A), we analyzed its mRNA and protein expression patterns in endometrial stem cells exposed or not to cigarette smoke, and SERPINB2 expression was found to be significantly and dose-dependently increased in endometrial stem cells after cigarette smoke exposure (Fig. 4B). We also used GEO metadata to further investigate relationships between toxic response and SERPINB2 expression and found SERPINB2 levels were markedly enhanced in response to many toxic stimuli, such as radiation, heavy metals, air pollution, and toxic chemicals (Fig. 4C). To assess whether aberrantly activated SERPINB2-related signaling pathways are associated with toxic responses, we investigated multiple gene expression patterns and their signaling networks using the web-based IPA (Ingenuity Pathway Analysis) platform. We found in lung cells, downstream mediators of SERPINB2, AGER, MUCI, and SETPD were activated after exposure to toxins (Fig. 4D), and in liver cells exposed to toxins, downstream mediators of SERPINB2, ALT and GOT were activated (Fig. 4E). These results suggest SERPINB2 level in human endometrial stem cells might be a marker of reproductive toxicity.

### SERPINB2 knockdown significantly abolishes the inhibitory effects of cigarette smoke on various tissue regeneration-associated functions

**Fig. 5:**
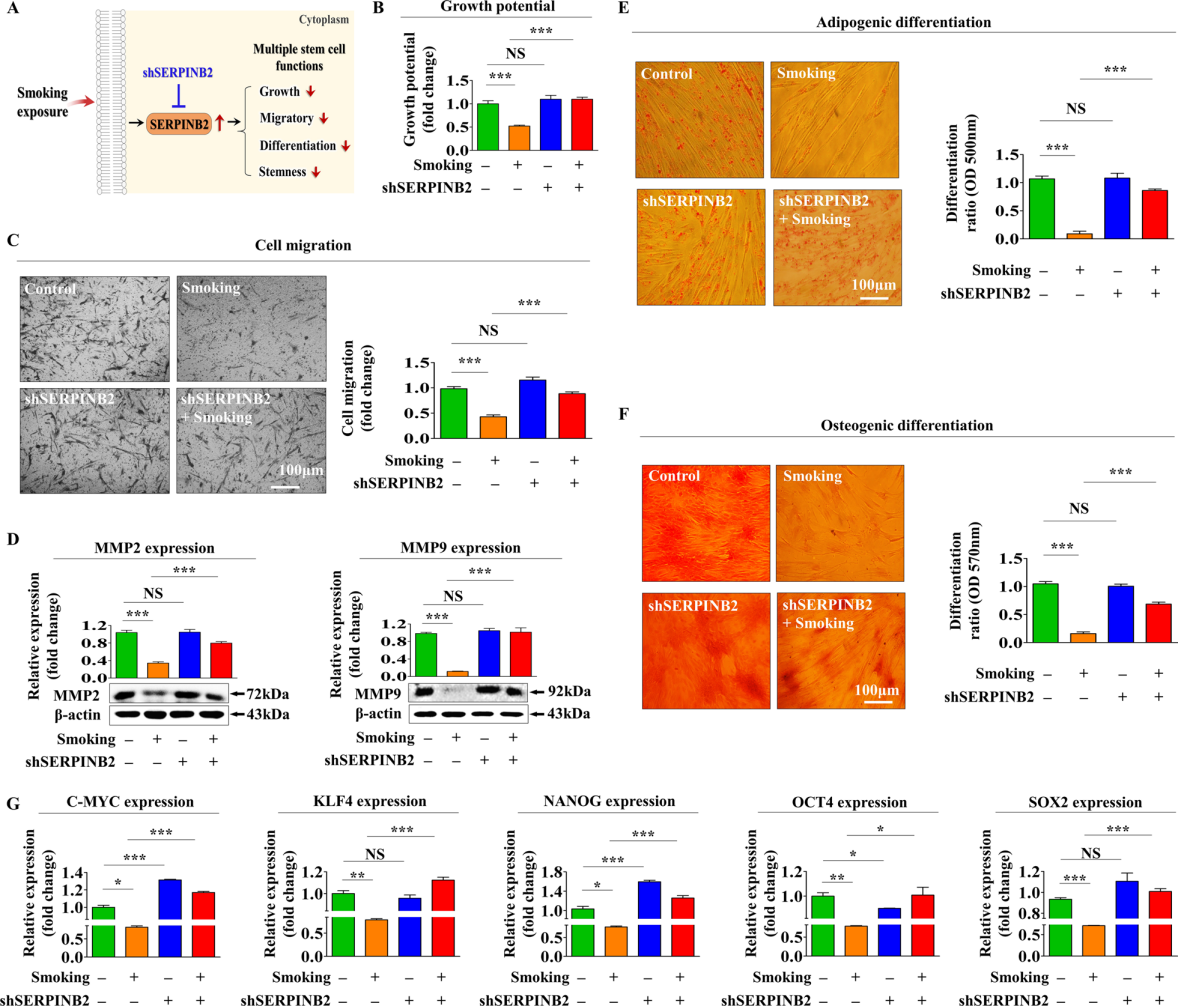
SERPINB2 mediated the harmful effects of cigarette smoke exposure on various tissue regeneration-associated functions of endometrial stem cells. Schematic diagram describing the roles of SERPINB2 as a key regulator that mediates cigarette smoke-induced harmful effects in endometrial stem cells (**A**). Cells were transfected with shRNA specifically targeting SERPINB2 and then exposed or not to 1% cigarette smoke extract for 72 h. Changes in cell viability were determined using an MTT assay (**B**). SERPINB2 knockdown attenuated cigarette smoke-mediated inhibition of endometrial stem cells migration as determined by transwell assay (**C**) and western blotting using MMP-2 and MMP-9 antibodies (**D**). Cells were transfected with a SERPINB2 shRNA and then treated with or without 1% cigarette smoke extract; subsequent changes in adipocyte (**E**) and osteoblast (**F**) differentiation abilities were analyzed by oil red O and alizarin red staining, respectively. Relative calcium mineral contents and lipid droplet formation within differentiated cells were assessed by measuring absorbance at 500 and 570 nm, respectively. Blocking of the effects of SERPINB2 knockdown on the cigarette smoke-induced inhibitions of pluripotency-associated factors C-MYC, KLF4, NANOG, OCT4, and SOX2 was investigated by real-time PCR (**G**). β-actin was used as the internal control, and PPIA as the housekeeping gene for real-time PCR analysis. All experiments were performed in triplicate. Data are presented as means ± SDs. *, *p* < 0.05; **, *p* < 0.005; and ***, *p* < 0.001 (two-sample *t* test)

To determine whether SERPINB2 mediates cigarette smoke-induced suppressive effects on various tissue regeneration-associated functions (Fig. 5A), SERPINB2 was knocked down by transfecting endometrial stem cells with specific shRNA targeting SERPINB2 (Additional file 1: Fig. S2A–C), and this abolished the cigarette smoke-induced suppressive effects on self-renewal ability (Fig. 5B), remarkably reduced its suppressive effects on migratory capacity (Fig. 5C), and prevented cigarette smoke-induced suppressions of MMP-2/9, which play crucial roles in cell invasion and migration (Fig. 5D). Furthermore, SERPINB2 knockdown significantly suppressed cigarette smoke-induced inhibitions of the adipocyte and osteoblast transdifferentiation**)** (Fig. 5E, F), and markedly reduced the inhibitory effects of cigarette smoke on the expressions of several pluripotency/stemness-related genes, C-MYC, KLF4, NANOG, OCT4, and SOX2 (Fig. 5G). These results indicate SERPINB2 might importantly attenuate cigarette smoke-induced suppressions of various tissue regeneration-associated functions of endometrial stem cells, such as self-renewal, migratory, pluripotency, and multilineage differentiation potential.

### SERPINB2 overexpression inhibits various regeneration capacity-related functions of endometrial stem cells

**Fig. 6:**
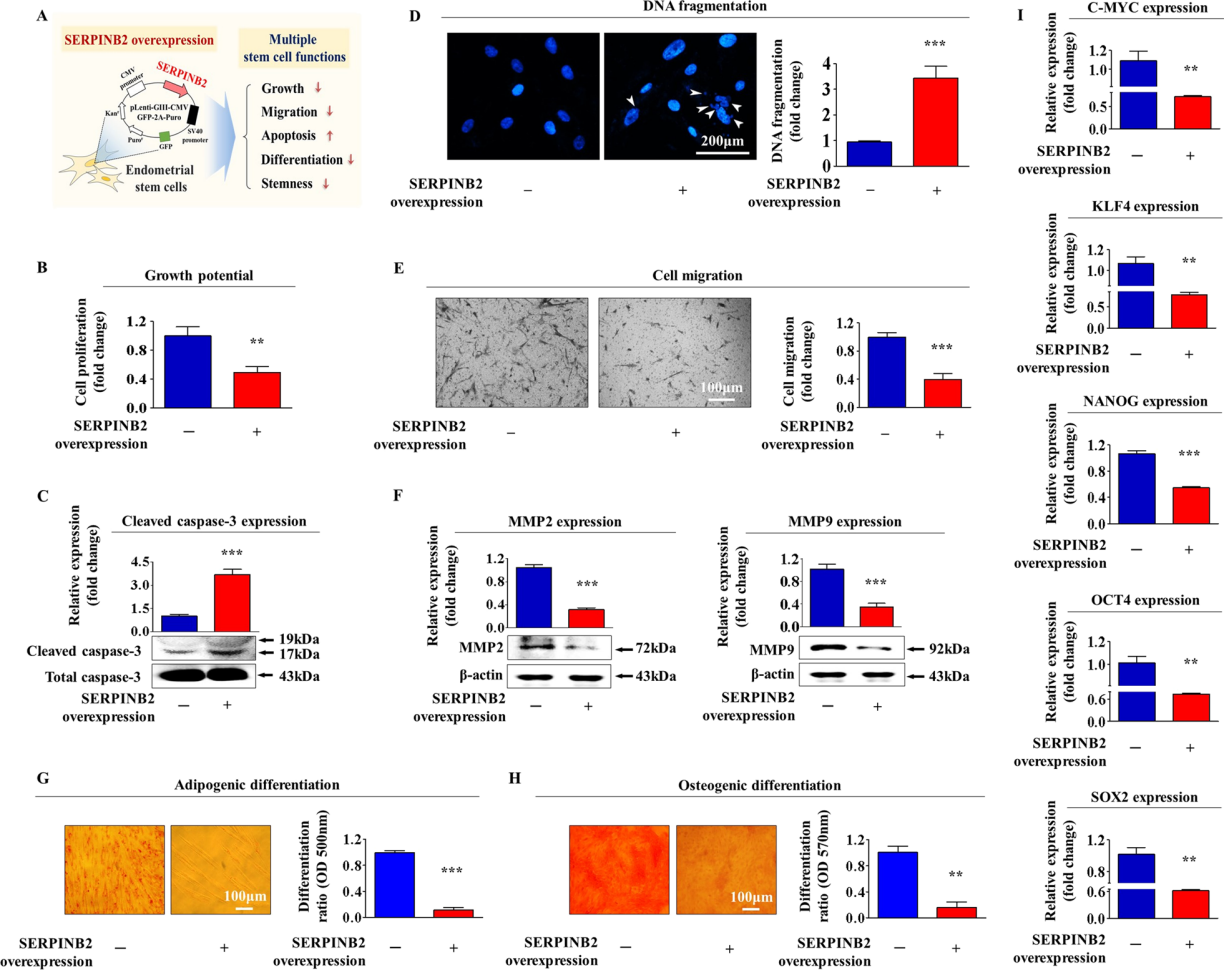
Effects of SERPINB2 overexpression on various tissue regeneration-associated functions of endometrial stem cells. Schematic showing the regulatory roles of SERPINB2 on various tissue regeneration-associated functions of endometrial stem cells (**A**). Transfection of endometrial stem cells with a specific retroviral expression vector for SERPINB2 significantly inhibition inhibited cell proliferation as compared with control vector transfection (**B**). Increased levels of caspase-3 activities following SERPINB2 overexpression were analyzed by western blotting (**C**), and SERPINB2 overexpression-induced apoptotic DNA fragmentations in endometrial stem cells were also analyzed by DAPI staining (**D**). The inhibitory effects of SERPINB2 overexpression on the migration capacity of endometrial stem cells were assessed by transwell assay (**E**) and western blotting using specific MMP-2 and MMP-9 antibodies (**F**). The inhibitory effects of SERPINB2 overexpression on osteogenic (**G**) and adipogenic (**H**) differentiation were analyzed by alizarin red or oil red O staining, respectively. Relative quantifications of calcium mineral contents and lipid droplet formation were determined by measuring absorbance at 570 or 500 nm, respectively. The inhibitory effects of SERPINB2 overexpression on the expressions of pluripotency-associated genes C-MYC, KLF4, NANOG, OCT4, and SOX2 were analyzed by real-time PCR (**I**). β-actin was used as an internal control, and PPIA as the housekeeping gene for real-time PCR analysis. All experiments were performed in triplicate. Data are presented as means ± SDs. *, *p* < 0.05; **, *p* < 0.005; and ***, *p* < 0.001 (two-sample *t* test)

To investigate whether SERPINB2 suppresses various regeneration capacity-related functions of endometrial stem cells (Fig. 6A), we overexpressed SERPINB2 in cells by transfecting them with a specific retroviral expression vector (Additional file 1: Fig. S3A, B). SERPINB2 overexpression was found to significantly reduce cell viabilities (Fig. 6B) and to significantly increase activated caspase-3 levels (Fig. 6C) and apoptotic DNA fragmentation (Fig. 6D), and transwell studies showed SERPINB2 overexpression significantly inhibited migratory capacity (Fig. 6E). Furthermore, western blot showed that SERPINB2 overexpression markedly reduced MMP-2 and MMP-9 levels (Fig. 6F) and significantly inhibited the ability to transdifferentiate to adipocytes (Fig. 6G) and osteoblasts (Fig. 6H). In addition, the mRNA levels of pluripotency/stemness-related genes, C-MYC, KLF4, NANOG, OCT4, and SOX2, were significantly reduced by SERPINB2 overexpression (Fig. 6I).

### Quantitative proteomic analysis of the inhibitory effects of cigarette smoke on various growth factors/cytokines and their signaling networks

**Fig. 7:**
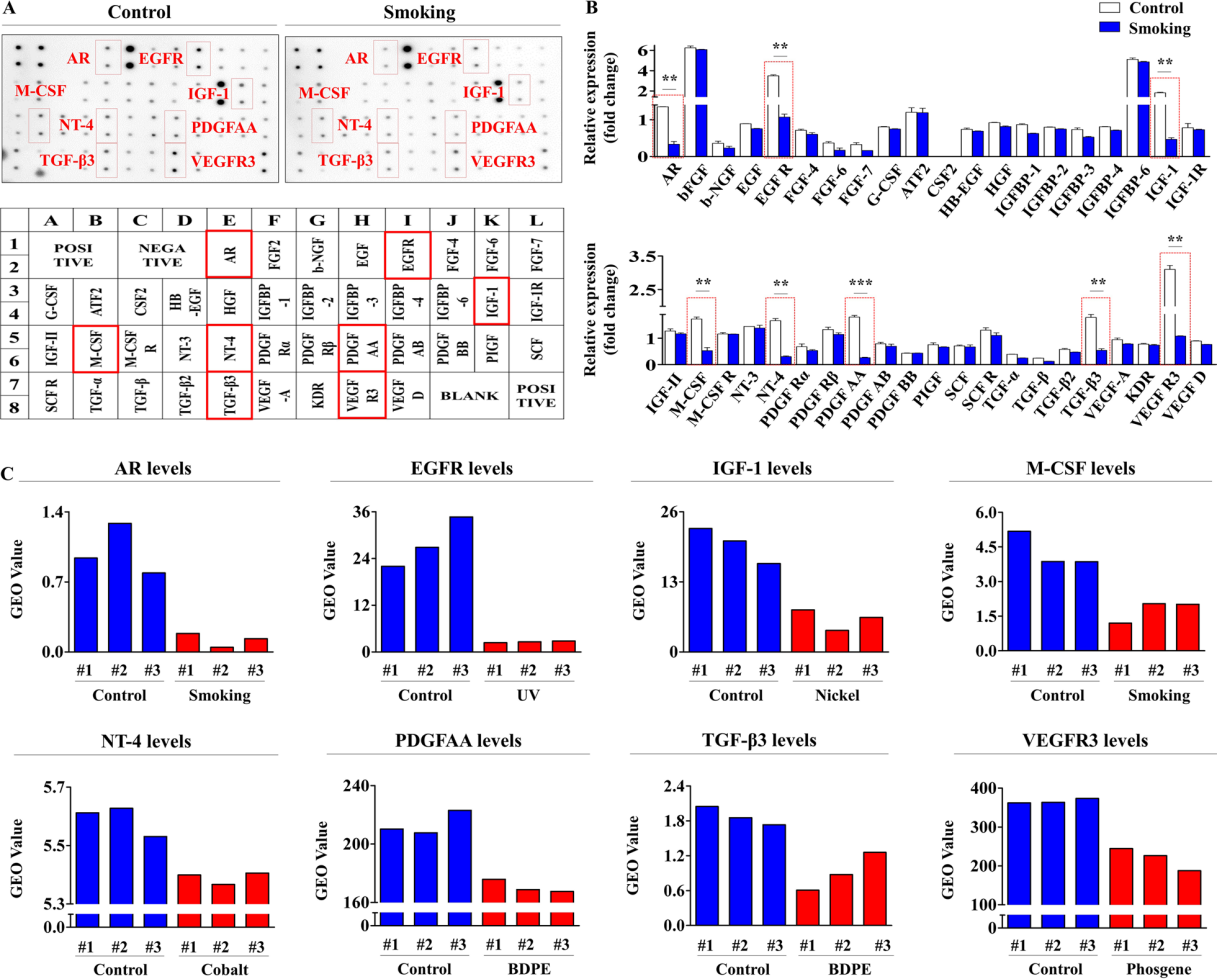
Cigarette smoke exposure significantly reduced the secretions of multiple cytokines and growth factors associated with toxic signaling networks. Antibody-based human growth factor arrays, in which the membrane was dotted with antibodies against 40 different proteins were used to detect cigarette smoke extract-induced changes in protein levels in conditioned media. The secretions of eight growth factors (AR, EGFR, IGF-1, M-CSF/CSF1, NT-4, PDGF-AA, TGFβ3, and VEGFR3) were markedly reduced by cigarette smoke extract (**A**, **B**). Relationships between these eight downregulated growth factors and exposure to toxicants were also assessed using the GEO database, which revealed the clear correlationship between the eight downregulated growth factors and exposure to various toxicants including smoking (**C**). Bar graphs represent the averages of three independent experiments. Significant differences are presented as *p < 0.05, **p < 0.005, and ***p < 0.001 (two-sample *t* test)

We postulated that cigarette smoke exposure could inhibit the secretion of multiple cytokines and/or growth factors, which in turn may be at least partly responsible for cigarette smoke-induced harmful effects on various tissue regeneration-associated functions of endometrial stem cells. Thus, to investigate the effects of cigarette smoke exposure on the secretions of growth factors or cytokines by endometrial stem cells, we investigated the effects of cigarette smoke on the secretion of multiple growth factors or cytokines using antibody array-based platform. The secretions of eight growth factors, namely androgen receptor (AR), epidermal growth factor receptor (EGFR), insulin-like growth factor-1 (IGF-1), macrophage colony-stimulating factor (M-CSF)/CSF1, neurotrophin-4 (NT-4), platelet-derived growth factor-AA (PDGF-AA), transforming growth factor β3 (TGFβ3), and vascular endothelial growth factor receptor 3 (VEGFR3), were found to be remarkably inhibited by cigarette smoke exposure (Fig. 7A, B). In addition, the GEO dataset showed that these prominent growth factors exhibit significant transcriptional changes after exposure to various toxic substances including cigarette smoke (Fig. 7C). Moreover, to assess whether these eight factors are associated with signaling pathways that control self-renewal ability, we analyzed the activation statuses of multiple signaling molecules and their related signaling networks using the web-based IPA platform. This analysis showed that positive regulators of AR (RELA, F7, and RICTOK), EGFR (EPAS1, SPP1, and HIF1A), CSF1 (ADORA3, C5, and AGER), and VEGFR3 (IFNG, TNF, and FOXO3) were suppressed (Additional file 1: Figs. S4A, B and S5A, B, respectively). In addition, we also investigated whether these prominent growth factors were related to signaling pathways regulating self-renewal ability, migratory capacity, or pluripotency/stemness using the Web-based genomic and data analysis platform GeneMANIA. To analyze functional interconnections, databases were filtered for based on multiple criteria, such as colocalization, genetic interactions, and co-expression. The results obtained suggested strong associations between these growth factors and essential cellular functions in terms of self-renewal ability, migratory capacity, and pluripotency/stemness (Additional file 1: Fig. S6A, B).

### Cigarette smoke exposure inhibits various tissue regeneration-associated functions of endometrial stem cells in vivo

**Fig. 8:**
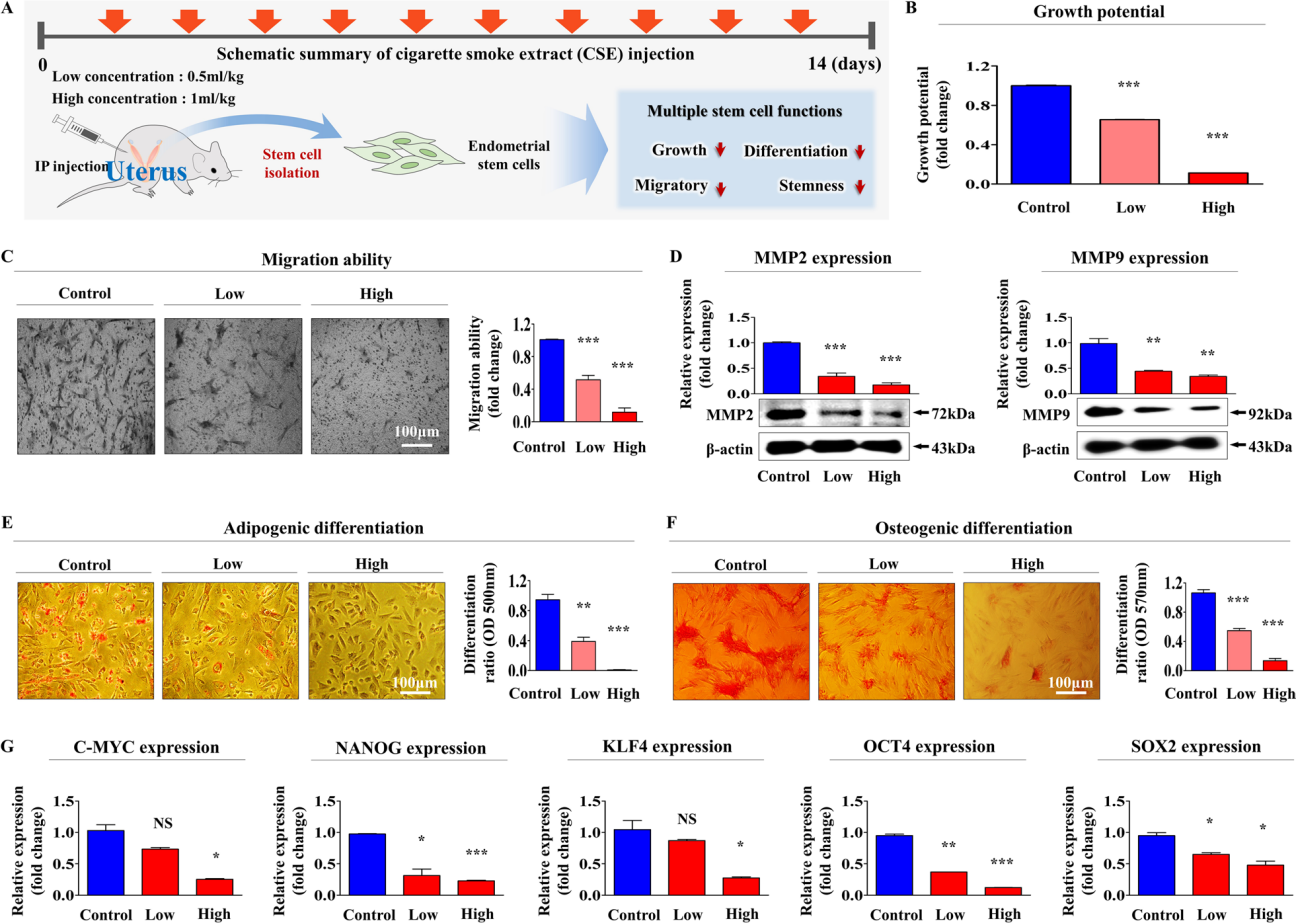
Cigarette smoke exposure markedly suppressed various tissue regeneration-associated functions of endometrial stem cells in vivo. Schematic of the in vivo experimental procedure used, which is described in detail in Materials and Methods (**A**). Mice were intraperitoneally administrated cigarette smoke extract 10 times at 0.5 or 1 mg/kg mg/kg for or vehicle. Mice endometrial stem cells were isolated from uterine tissues, and the inhibitory effects of cigarette smoke on cell viability were assessed using an MTT assay (**B**). The changes in stem cell migratory abilities were analyzed using a transwell assay (**C**) and by western blotting using MMP-2 and MMP-9 antibodies (**D**). The inhibitory effects of cigarette smoke exposure on adipocyte (**E**) and osteoblast (**F**) differentiation of mouse endometrial stem cells in vivo were analyzed by oil red O and alizarin red staining, respectively. Relative quantifications of calcium mineral content and lipid droplet formation within differentiated cells were evaluated by measuring absorbance at 500 and 570 nm, respectively. The inhibitory effects of cigarette smoke exposure on the expression of various pluripotency-associated genes, C-MYC, KLF4, NANOG, OCT4, and SOX2 were analyzed by real-time PCR (**G**). β-actin was used as the internal control, and PPIA as the housekeeping gene for real-time PCR. All experiments were performed in triplicate. Data are presented as means ± SDs. *, *p* < 0.05; **, *p* < 0.005; and ***, *p* < 0.001 (two-sample *t* test)

Our in vitro data also indicated that cigarette smoke exposure may inhibit the tissue regeneration-associated functions of resident endometrial stem cells in vivo. Therefore, we investigated whether cigarette smoke exposure exerted similar effects in an animal model. Mice were injected intraperitoneally with low (0.5 mg/kg) or high (1 mg/kg) doses of cigarette smoke extract (10 times for 2 weeks), and endometrial stem cells were then isolated from mice uterine tissues (Fig. 8A). Consistent with our in vitro results, cigarette smoke exposure significantly inhibited the self-renewal potential of mouse endometrial stem cells in vivo in a dose-dependent manner (Fig. 8B). In addition, transwell assay results showed cigarette smoke exposure suppressed the migration capacity of mice endometrial stem cells (Fig. 8C)*,* and Western blotting for MMP-2 and MMP-9, which are required for cell migration, showed that cigarette smoke exposure suppressed their intracellular levels (Fig. 8D). Notably, cigarette smoke exposure also significantly inhibited the multilineage differentiation capacity of mice endometrial stem cells into adipocytes (Fig. 8E) and osteoblasts (Fig. 8F) in vivo, and levels of the pluripotency/stemness-associated genes C-MYC, NANOG, KLF4, OCT4, and SOX2 were remarkably decreased (Fig. 8G). We also investigated the effects of cigarette smoke exposure on the tissue regeneration-associated functions of other types of stem cells, such as bone marrow- and adipose tissue-derived stem cells. Mice were injected intraperitoneally with low (0.5 mg/kg) or high (1 mg/kg) doses of cigarette smoke (10 times for 2 weeks), and resident stem cells were isolated from adipose tissues (Additional file 1: Fig. S7A) and bone marrow (Additional file 1: Fig. S8A). Consistently, cigarette smoke exposure was found to remarkably reduce the self-renewal (Additional file 1: Figs. S7B and S8B), migratory (Additional file 1: Figs. S7C, D and S8C, D), multileague differentiation capacities (Additional file 1: Figs. S7E, F and S8E, F), and pluripotency/stemness-associated genes (Additional file 1: Figs. S7G and S8G) in these stem cells, which concurred with our in vitro results. Taken together, these observations indicate that cigarette smoke exposure suppresses the tissue regeneration-associated functions of various types of stem cells.

## Discussion

Cigarette smoke extracts have been widely used for various in vitro and in vivo toxicity studies on smoking, and the results obtained closely match those obtained using exhaled mainstream smoke under controlled conditions [59]. Previous studies have shown that 1% cigarette smoke extract approximates to human pulmonary artery endothelial cell (HPAEC) exposure in people that smoke slightly less than two packs of cigarettes per day [60, 61]. Therefore, we postulated that 0.5% or 1% concentrations of cigarette smoke extract would mimic the exposure experienced by regular smokers. However, the relation between lung endothelial cell exposure and uterine endometrial stem cell exposure has not been established. Furthermore, relatively little is known of the direct effects of smoking on human endometrial cellular components, including stem cells, or the molecular mechanisms involved.

The entire endometrial surface (lining of the uterine cavity) is restored for the next reproductive cycle immediately after menstruation, [62] and this unique regenerative ability of endometrium is essential for successful embryo implantation and subsequent pregnancy outcomes [62, 63]. Endometrial stem cells within the basal layer (stratum basalis), adjacent to myometrium, are not shed during menstrual cycles, because they are primarily responsible for the regenerative capacity of endometrium [64]. Lucas et al. observed that the impaired proliferative capacity of endometrial stem cells within uterine tissue is closely related to increased miscarriage rates and infertility [16]. Significant reduction in the proliferative activity of endometrial stem cells was observed in ~ 40% of patients with consecutive pregnancy loss as compared with only 10% in women that experienced successful pregnancies [16]. Increased cellular senescence (aging) of endometrial stem cells facilitates inflammation-induced autoimmune response, and this may be closely related to consecutive pregnancy loss [65]. Because of this close relationship between endometrial receptivity and the quality of tissue resident endometrial stem cells, intensive investigation of endometrial stem cells may provide novel insights into the major causes of recent increases in environmental factors-related infertility. In particular, challenging questions are being asked about the possible direct negative impacts of cigarette smoking on endometrial stem cell quality and female infertility. Few studies have examined the direct effects of cigarette smoking on human endometrial cell functions, though it has been reported cigarette smoke exposure negatively affects cyclic regeneration of endometrium during the menstrual cycle by disrupting the activities of various angiogenesis- and decidualization-associated factors [66]. Cigarette smoke has also been shown to induce cellular stress, inflammation, and endometrial stromal cell remodeling through HIF-1α signaling [67], and Omid et al. [68] observed that cigarette smoke exposure significantly inhibits the self-renewal capacities of uterine epithelial cells through a nitric oxide-mediated signaling pathway. In addition to these effects on the endometrium, several studies have revealed that high concentrations of nicotine can induce apoptotic cell death of ovarian follicles, which lead to increased infertility in women smoking cigarette [69, 70]. However, the potential direct effects of cigarette smoking on endometrial stem cells, which are critical for endometrial receptivity and subsequent pregnancy rates, have not been investigated. In this context, we suggest cigarette smoking directly can reduce endometrial receptivity by inhibiting various tissue regeneration-associated functions of endometrial stem cells, and thus, result in adverse pregnancy outcomes. Although cigarette smoke contains more than 3000 different chemicals, nicotine is the major active component of cigarette that causes various harmful effects via its specific interaction to nicotinic acetylcholine receptor channels on the cell membrane [71, 72]. Interestingly, Arredondo et al. also observed that nicotine exposure normal human oral keratinocytes can alter the ligand-binding kinetics of nicotinic acetylcholine receptor, indicating structural changes of the receptors at both the mRNA and protein levels [73].

Consistent with this hypothesis, the present study shows that cigarette smoke exposure remarkably suppresses various tissue regeneration-associated functions of endometrial stem cells, such as the clonogenicity, cellular senescence, migratory ability, transdifferentiation potential, pluripotent/stemness, in vitro and in vivo and metabolic features in vivo (Figs. 1, 2, 3 and 8A-G, respectively). Furthermore, cigarette smoke exposure significantly decreased the secretions of eight growth factors, namely AR, EGFR, IGF-1, M-CSF/CSF1, NT-4, PDGF-AA, TGFβ3, and VEGFR3 (Fig. 7A, B). The GEO metadata repository dataset showed that the expressions of these eight prominent growth factors in endometrial stem cells underwent similar changes after exposure to cigarette smoke or various toxic substances including smoking (Fig. 7C). These results indicate cigarette smoke exposure has suppressive effects on various endometrial stem cell functions and that these effects are due, at least in part, to reduced secretions of multiple growth factors and cytokines that regulate various essential cellular functions, such as growth, migration, pluripotency/stemness, and apoptosis. Furthermore, we observed cigarette smoke-induced suppressions of endometrial stem cell proliferation (Fig. 5B), migration (Fig. 5C, D), multilineage differentiation capacity (Fig. 5E, F), and pluripotency/stemness (Fig. 5G) were significantly abolished by SERPINB2 depletion. Indeed, SERPINB2 overexpression also inhibited various regeneration capacity-related functions of endometrial stem cells (Fig. 6A–I). These results suggest that SERPINB2 functions as a key mediator of the harmful effects of cigarette smoking on the tissue regeneration-associated functions of endometrial stem cells. Similarly, aberrant activation of SERPINB2 has been reported to be significantly associated with tumor progression (metastasis) and poor prognosis in various cancer types, such as endometrial [74], colorectal [75], ovarian [76], and bladder [77] cancers. Moreover, Qin Hu et al. observed that dioxin (TCDD) exposure markedly increased SERPINB2 expression levels in human skin cells (keratinocytes) [78]. These previous studies suggest that SERPINB2 may function as a crucial mediator of various toxic responses. In addition, we recently found that enhanced SERPINB2 expression significantly suppressed the self-renewal capacity, migration potential, transdifferentiation ability, and pluripotency/stemness of human umbilical cord blood-derived mesenchymal stem cells [27].

## Conclusion

Taken together, our findings suggest cigarette smoking directly reduces endometrial receptivity by suppressing various tissue regeneration-associated functions of endometrial stem cells. To the best of our knowledge, this is the first study to investigate the harmful effects of cigarette smoking on the regenerative potential of endometrial stem cells and to provide clear evidence that SERPINB2 may function as a crucial regulator of the harmful effects of smoking on human endometrial stem cells. We believe the present study increases understanding of the molecular mechanisms underlying the associations between cigarette smoking and female infertility and miscarriage rates and, hopefully, will encourage researchers developing novel therapeutic approaches aimed at enhancing the reproductive rates of subfertile or infertile women.

## Supplementary Information

**Additional file 1: Fig. S1.** Establishment of human endometrial stem cells from uterine endometrial tissues. Isolated human endometrial stem cells based were observed under a phase-contrast microscope to assess their overall morphological features (**a**). Isolated human endometrial stem cells were positive for various stemness-associated antigens (CD44, CD73, CD105, CD140b, CD146, and susD2) and negative for multiple hematopoietic stem cell lineage antigens (CD34 and CD45) (**b**). Differentiation into adipocyte and osteoblast was analyzed by oil red O staining and alizarin red staining, respectively. Relative quantifications of calcium mineral contents and lipid droplet formation within differentiated cells were performed by measuring absorbance at 500 and 570 nm, respectively (**c**). All experiments were performed in triplicate. Data are presented as mean ± SDs. *, *p*< 0.05; **, *p*< 0.005; and ***, *p*< 0.001 (two-sample t test). **Fig. S2.** Efficacy of SERPINB2 knockdown using specific shRNAs in endometrial stem cells. Human endometrial stem cells were transfected with multiple shRNAs (#1, #2, #3, #5, or #4), which specifically target SERPINB2, or with a non-targeting control shRNA (**a**). shRNA construct #2 (hereafter referred to as SERPINB2 shRNA) most effectively knocked downSERPINB2 at the mRNA (**b**) and protein levels (**c**). β-actin was used as the internal control, and PPIA was used as a housekeeping gene for real-time PCR. All experiments were performed in triplicate. Data are presented as means ± SDs. *, *p*< 0.05; **, *p*< 0.005; and ***, *p*< 0.001 (two-sample *t* test). **Fig. S3.** Efficiency of inducing SERPINB2 overexpression using a specific retroviral expression vector. Endometrial stem cells were specifically transfected with a retroviral expression vector for SERPINB2 (**a**). Successful SERPINB2 overexpression was confirmed at the mRNA (**b**)and protein (**c**)levels. β-actin was used as internal control, and PPIA as the housekeeping gene for real-time PCR. All experiments were performed in triplicate. Data are presented as mean ± SDs. *, *p*< 0.05; **, *p*< 0.005; and ***, *p*< 0.001 (two-sample *t* test). **Fig. S4.** The cigarette smoke-induced inhibitory effects on various growth factor secretions are closely interacted with self-renewal capacity-associated signaling pathways. Differentially activated signaling molecules secreted from toxic substance-exposed and non-exposed cells were analyzed using IPA software to predict their activation states (activated or inhibited) with respect to AR (GSE69851) (**a**) and EGFR(GSE60408) (**b**) associated signaling integrities. **Fig. S5.** The cigarette smoke-induced inhibitory effects on various growth factor secretions are closely interacted with self-renewal capacity-associated signaling pathways. Differentially activated signaling molecules in toxic substance-exposed cells and non-exposed cells were analyzed using IPA software to predict their activation states (activated or inhibited) with respect to CSF-1 (GSE69851) (**a**) or VEGFR3 (GSE60408) (**b**)associated signaling integrities. **Fig. S6.** Functional interactions between cigarette smoke-induced factors and signaling networks that regulate multiple essential cellular functions. Signaling network analysis was conducted using GeneMANIA (http://www.genemania.org) to analyze interactions between the cigarette smoke-induced changes of eight growth factors (AR, EGFR, IGF-1, M-CSF/CSF1, NT-4, PDGF-AA, TGFβ3, and VEGFR3) and signaling networks that regulate self-renewal, pluripotency/stemness, or migratory capacity. The results showed strong interaction between these eight prominent growth factors and various essential cellular functions, such as proliferative capacity, pluripotency/stemness, and migration potential (**a**, **b**). **Fig. S7.** Cigarette smoke exposure markedly suppressed various tissue regeneration-associated functions of adipose tissue-derived stem cells in vivo. Schematic of the in vivo experimental procedure described in Materials and Methods (**a**). Mice were intraperitoneally administrated a low (0.5 mg/kg) or high (1 mg/kg) dose of cigarette smoke extract or vehicle (medium) 10 times. Adipose tissue-derived stem cells were then isolated from mouse adipose tissues, and the inhibitory effects of cigarette smoke exposure on cell viability were assessed using an MTT assay (**b**). The changes in stem cell migratory abilities were determined using a transwell assay (**c**)and by western blotting using MMP-2 and MMP-9 antibodies (**d**). The inhibitory effects of cigarette smoke extract on the differentiation of mouse adipose tissue-derived stem cells into adipocytes (**e**) and osteoblast (**f**) in vivo were analyzed by oil red O and alizarin red staining, respectively. Relative quantifications of calcium mineral contents and lipid droplet formation within differentiated cells were evaluated by measuring absorbance at 500 and 570 nm, respectively. The inhibitory effects of cigarette smoke exposure on the expressions of various pluripotency-associated genes, C-MYC, KLF4, NANOG, OCT4, and SOX2, were determined by real-time PCR (**g**). β-actin was used as the internal control, and PPIA as the housekeeping gene for real-time PCR analysis. All experiments were performed in triplicate. Data are presented as means ± SDs. *, *p*< 0.05; **, *p*< 0.005; and ***, *p*< 0.001 (by the two-sample *t* test). **Fig. S8.** Cigarette smoke exposure markedly suppressed various tissue regeneration-associated functions of bone marrow-derived stem cells in vivo. Schematic of the in vivo experimental procedure described in Materials and Methods (**a**). Mice were intraperitoneally administrated low (0.5 mg/kg) or high (1 mg/kg) dose of cigarette smoke extract 10 times or vehicle (medium). Bone marrow-derived stem cells were isolated from bone marrow, and the inhibitory effects of cigarette smoke exposure on cell viability were assessed using an MTT assay (**b**). The changes in stem cell migratory abilities were analyzed using a transwell assay (**c**)and western blotting was performed using MMP-2 and MMP-9 antibodies (**d**). The inhibitory effects of cigarette smoke exposure on the differentiations of mouse bone marrow-derived stem cells into adipocytes (**e**) or osteoblasts (**f**) in vivo were assessed by oil red O and alizarin red staining, respectively. Relative quantifications of calcium mineral contents and lipid droplet formation within differentiated cells were performed by measuring absorbance at 500 and 570 nm, respectively. The inhibitory effects of cigarette smoke exposure on the expressions of various pluripotency-associated genes, C-MYC, KLF4, NANOG, OCT4, and SOX2, were analyzed by real-time PCR (**g**). β-actin was used as the internal control, and PPIA as the housekeeping gene for real-time PCR analysis. All experiments were performed in triplicate. Data are presented as means ± SDs. *, *p*< 0.05; **, *p*< 0.005; and ***, *p*< 0.001 (two-sample *t* test).

## Data Availability

The data and material support that all findings could be found.

## Abbreviations

AR: Androgen receptor
CS: Cigarette smoke
ECARs: Extracellular acidification rates
EGFR: Epidermal growth factor receptor
GEO: Gene expression omnibus
HIF-1α: Hypoxia-inducible factor 1α
HPAEC: Human pulmonary artery endothelial cell
IGF-1: Insulin-like growth factor-1
IPA: Ingenuity pathway analysis
M-CSF: Macrophage colony-stimulating factor
MMP-2: Matrix metalloproteinase-2
MMP-9: Matrix metalloproteinase-9
NT-4: Neurotrophin-4
OCR: Oxygen consumption rates
PDGF-AA: Platelet-derived growth factor-AA
SA-β-Gal: Senescence-associated β-galactosidase
TGFβ3: Transforming growth factor β3
VEGFR3: Vascular endothelial growth factor receptor 3
2-DG: 2-Deoxyglucose
