# Supplementary material for: Effects of smoking on the tissue regeneration-associated functions of human endometrial stem cells via a novel target gene SERPINB2
Source: Stem Cell Res Ther. 2022 Aug 5;13:404. doi: 10.1186/s13287-022-03061-1 (PMC9356492; doi:10.1186/s13287-022-03061-1)
Supplement: Supplementary file 1 — Additional file 1: Fig. S1. Establishment of human endometrial stem cells from uterine endometrial tissues. Isolated human endometrial stem cells based were observed under a phase-contrast microscope to assess their overall morphological features (a). Isolated human endometrial stem cells were positive for various stemness-associated antigens (CD44, CD73, CD105, CD140b, CD146, and susD2) and negative for multiple hematopoietic stem cell lineage antigens (CD34 and CD45) (b). Differentiation into adipocyte and osteoblast was analyzed by oil red O staining and alizarin red staining, respectively. Relative quantifications of calcium mineral contents and lipid droplet formation within differentiated cells were performed by measuring absorbance at 500 and 570 nm, respectively (c). All experiments were performed in triplicate. Data are presented as mean ± SDs. *, p< 0.05; **, p< 0.005; and ***, p< 0.001 (two-sample t test). Fig. S2. Efficacy of SERPINB2 knockdown using specific shRNAs in endometrial stem cells. Human endometrial stem cells were transfected with multiple shRNAs (#1, #2, #3, #5, or #4), which specifically target SERPINB2, or with a non-targeting control shRNA (a). shRNA construct #2 (hereafter referred to as SERPINB2 shRNA) most effectively knocked downSERPINB2 at the mRNA (b) and protein levels (c). β-actin was used as the internal control, and PPIA was used as a housekeeping gene for real-time PCR. All experiments were performed in triplicate. Data are presented as means ± SDs. *, p< 0.05; **, p< 0.005; and ***, p< 0.001 (two-sample t test). Fig. S3. Efficiency of inducing SERPINB2 overexpression using a specific retroviral expression vector. Endometrial stem cells were specifically transfected with a retroviral expression vector for SERPINB2 (a). Successful SERPINB2 overexpression was confirmed at the mRNA (b)and protein (c)levels. β-actin was used as internal control, and PPIA as the housekeeping gene for real-time PCR. All experiments were performed in [file 13287_2022_3061_MOESM1_ESM.pdf]

Supplementary Fig. 1

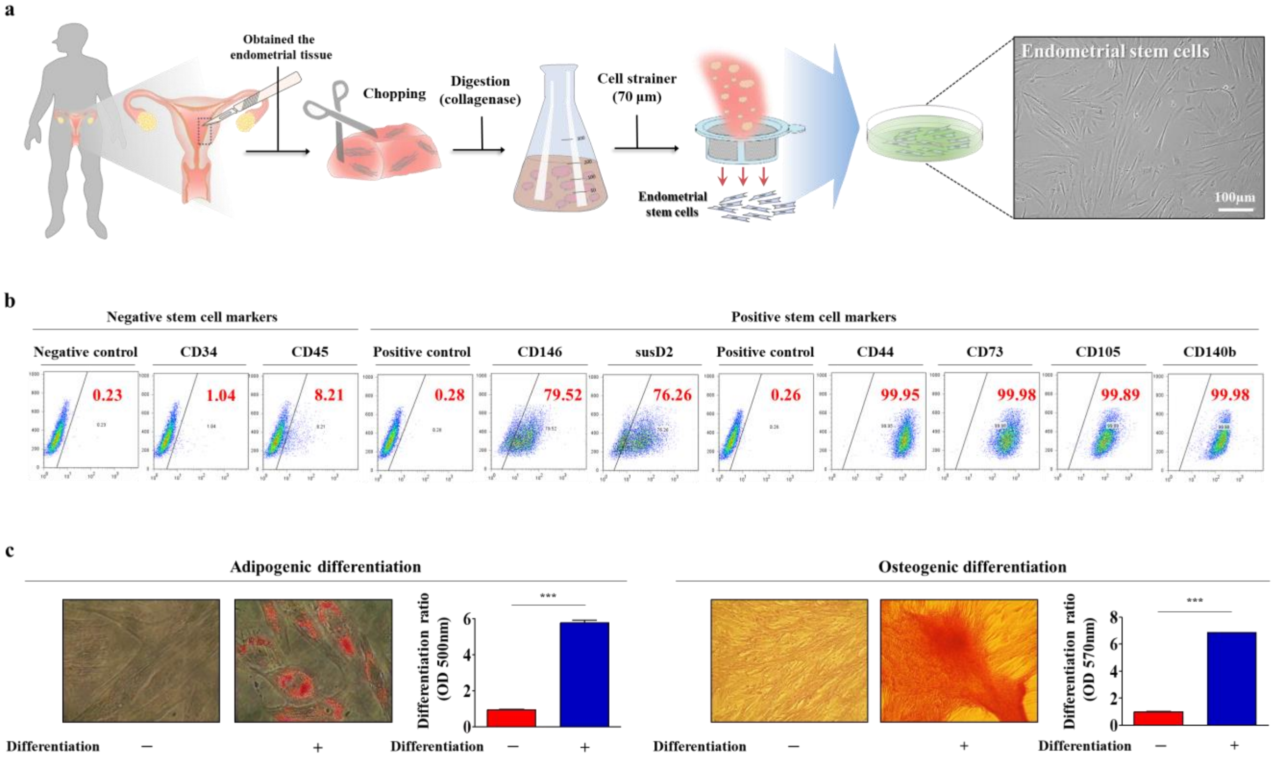

Supplementary fig. 1 Establishment of human endometrial stem cells from uterine endometrial tissues. Isolated human endometrial stem cells based were observed under a phase-contrast microscope to assess their overall morphological features **(a)**. Isolated human endometrial stem cells were positive for various stemness-associated antigens (CD44, CD73, CD105, CD140b, CD146, and susD2) and negative for multiple hematopoietic stem cell lineage antigens (CD34 and CD45) **(b)**. Differentiation into adipocyte and osteoblast was analyzed by oil red O staining and alizarin red staining, respectively. Relative quantifications of calcium mineral contents and lipid droplet formation within differentiated cells were performed by measuring absorbance at 500 and 570 nm, respectively **(c)**. All experiments were performed in triplicate. Data are presented as mean ± SDs. *, $p < 0.05$; **, $p < 0.005$; and ***, $p < 0.001$ (two-sample t-test).

Supplementary Fig. 2

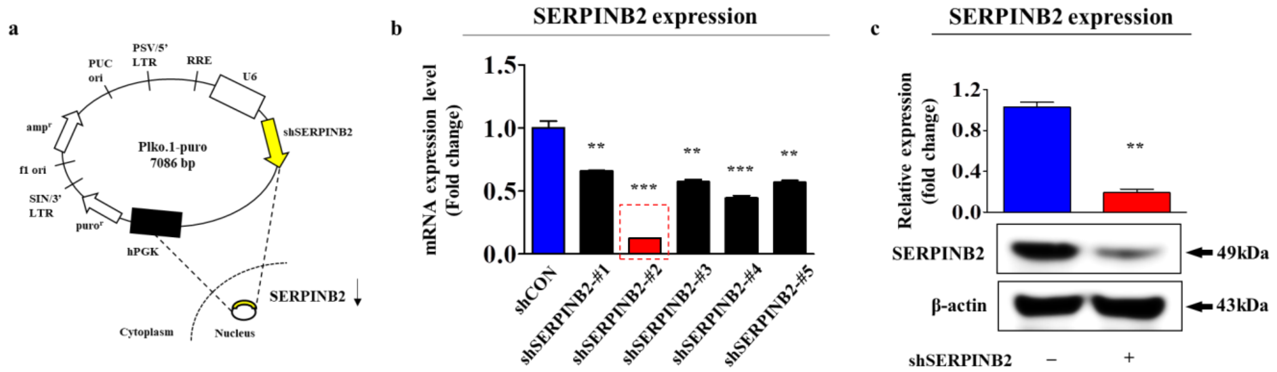

Supplementary fig. 2 Efficacy of SERPINB2 knockdown using specific shRNAs in endometrial stem cells. Human endometrial stem cells were transfected with multiple shRNAs (#1, #2, #3, #5, or #4), which specifically target SERPINB2, or with a non-targeting control shRNA **(a)**. shRNA construct #2 (hereafter referred to as SERPINB2 shRNA) most effectively knocked down SERPINB2 at the mRNA **(b)** and protein levels **(c)**. β-actin was used as the internal control, and PPIA was used as a housekeeping gene for real-time PCR. All experiments were performed in triplicate. Data are presented as means ± SDs. *, $p < 0.05$; **, $p < 0.005$; and ***, $p < 0.001$ (two-sample t-test).

Supplementary Fig. 3

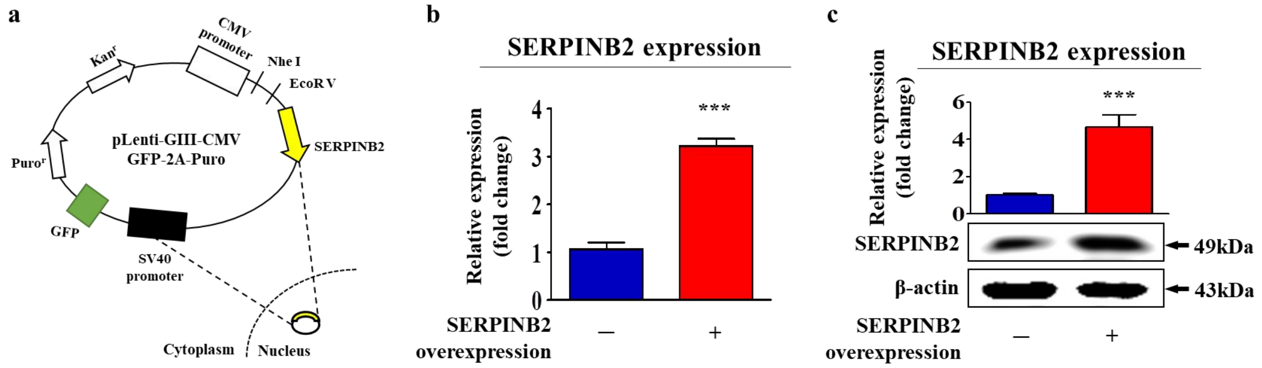

Supplementary fig. 3 Efficiency of inducing SERPINB2 overexpression using a specific retroviral expression vector. Endometrial stem cells were specifically transfected with a retroviral expression vector for SERPINB2 **(a)**. Successful SERPINB2 overexpression was confirmed at the mRNA **(b)** and protein **(c)** levels. β-actin was used as internal control, and PPIA as the housekeeping gene for real-time PCR. All experiments were performed in triplicate. Data are presented as mean ± SDs. *, $p < 0.05$; **, $p < 0.005$; and ***, $p < 0.001$ (two-sample t-test).

Supplementary Fig. 4

a

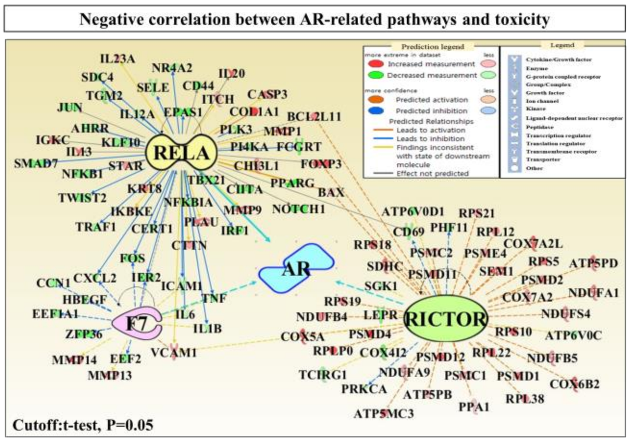

Signaling pathways	Predicted activation group	# of Target molecules in dataset	Predicted activation state	Activation z-score	P-value of overlap
**RELA**	Toxicant treated cells (vs non-treated cells)	155	Inhibition	-3.570	2.08E-02
**F7**	Toxicant treated cells (vs non-treated cells)	14	Inhibition	-2.688	2.45E-01
**RICTOR**	Toxicant treated cells (vs non-treated cells)	118	Inhibition	-7.575	4.69E-06

b

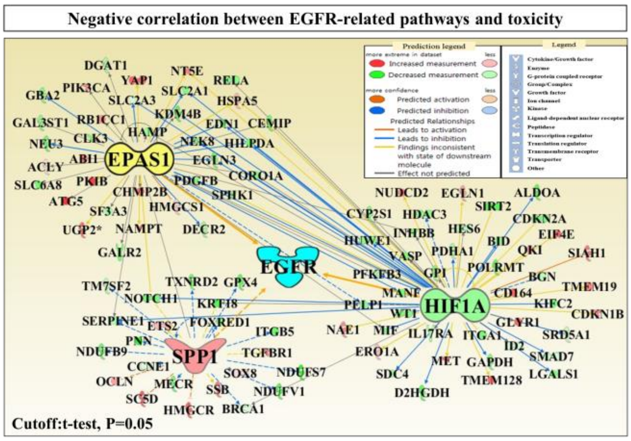

Signaling pathways	Predicted activation group	# of Target molecules in dataset	Predicted activation state	Activation z-score	P-value of overlap
**EPAS1**	Toxicant treated cells (vs non-treated cells)	42	Inhibition	-1.798	1.29E-02
**SPP1**	Toxicant treated cells (vs non-treated cells)	23	Inhibition	-2.681	100E00
**HIF1A**	Toxicant treated cells (vs non-treated cells)	64	Inhibition	-1.835	1.71E-01

Supplementary fig. 4 The cigarette smoke-induced inhibitory effects on various growth factor secretions are closely interacted with self-renewal capacity-associated signaling pathways. Differentially activated signaling molecules secreted from toxic substance-exposed and non-exposed cells were analyzed using IPA software to predict their activation states (activated or inhibited) with respect to AR (GSE69851) **(a)** and EGFR (GSE60408) **(b)** associated signaling integrities.

Supplementary Fig. 5

a

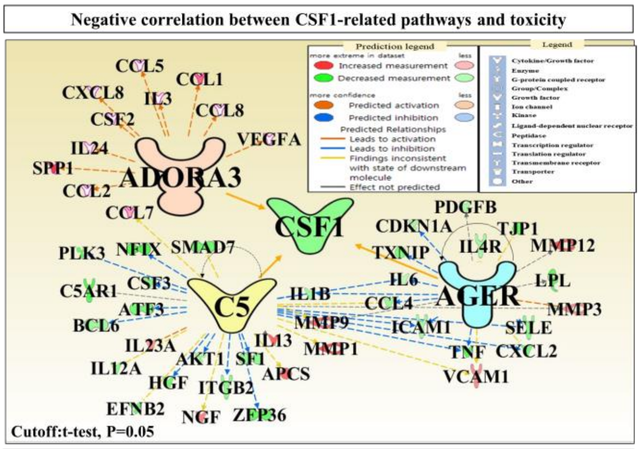

Signaling pathways	Predicted activation group	# of Target molecules in dataset	Predicted activation state	Activation z-score	P-value of overlap
**ADORA3**	Toxicant treated cells (vs non-treated cells)	13	Inhibition	-0.832	2.28E-02
**C5**	Toxicant treated cells (vs non-treated cells)	46	Inhibition	-2.225	9.83E-02
**AGER**	Toxicant treated cells (vs non-treated cells)	20	Inhibition	-1.915	1.00E00

b

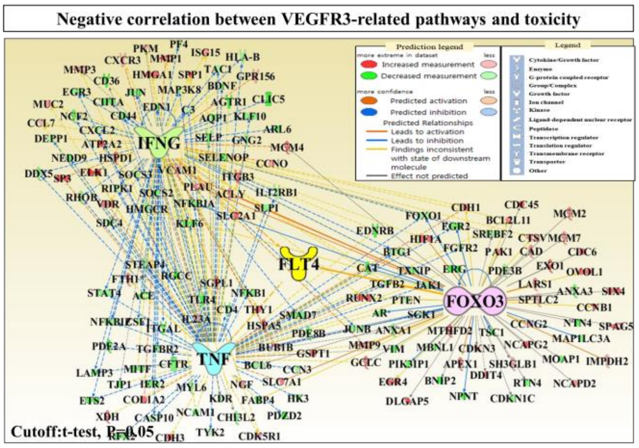

Signaling pathways	Predicted activation group	# of Target molecules in dataset	Predicted activation state	Activation z-score	P-value of overlap
**IFNG**	Toxicant treated cells (vs non-treated cells)	507	Inhibition	-3.188	1.80E-06
**TNF**	Toxicant treated cells (vs non-treated cells)	676	Inhibition	-1.029	1.60E-08
**FOXO3**	Toxicant treated cells (vs non-treated cells)	161	Inhibition	-2.175	5.71E-12

Supplementary fig. 5 The cigarette smoke-induced inhibitory effects on various growth factor secretions are closely interacted with self-renewal capacity-associated signaling pathways. Differentially activated signaling molecules in toxic substance-exposed cells and non-exposed cells were analyzed using IPA software to predict their activation states (activated or inhibited) with respect to CSF-1 (GSE69851) **(a)** or VEGFR3 (GSE60408) **(b)** associated signaling integrities.

Supplementary Fig. 6

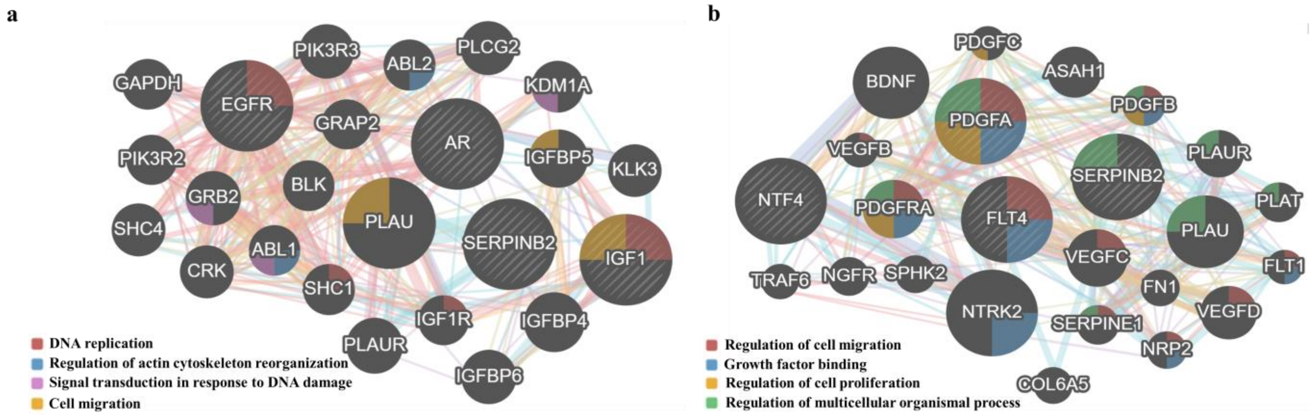

Supplementary fig 6 Functional interactions between cigarette smoke-induced factors and signaling networks that regulate multiple essential cellular functions. Signaling network analysis was conducted using GeneMANIA (http://www.genemania.org) to analyze interactions between the cigarette smoke-induced changes of eight growth factors (AR, EGFR, IGF-1, M-CSF/CSF1, NT-4, PDGF-AA, TGFβ3, and VEGFR3) and signaling networks that regulate self-renewal, pluripotency/stemness, or migratory capacity. The results showed strong interaction between these eight prominent growth factors and various essential cellular functions, such as proliferative capacity, pluripotency/stemness, and migration potential **(a-b)**.

Supplementary Fig. 7

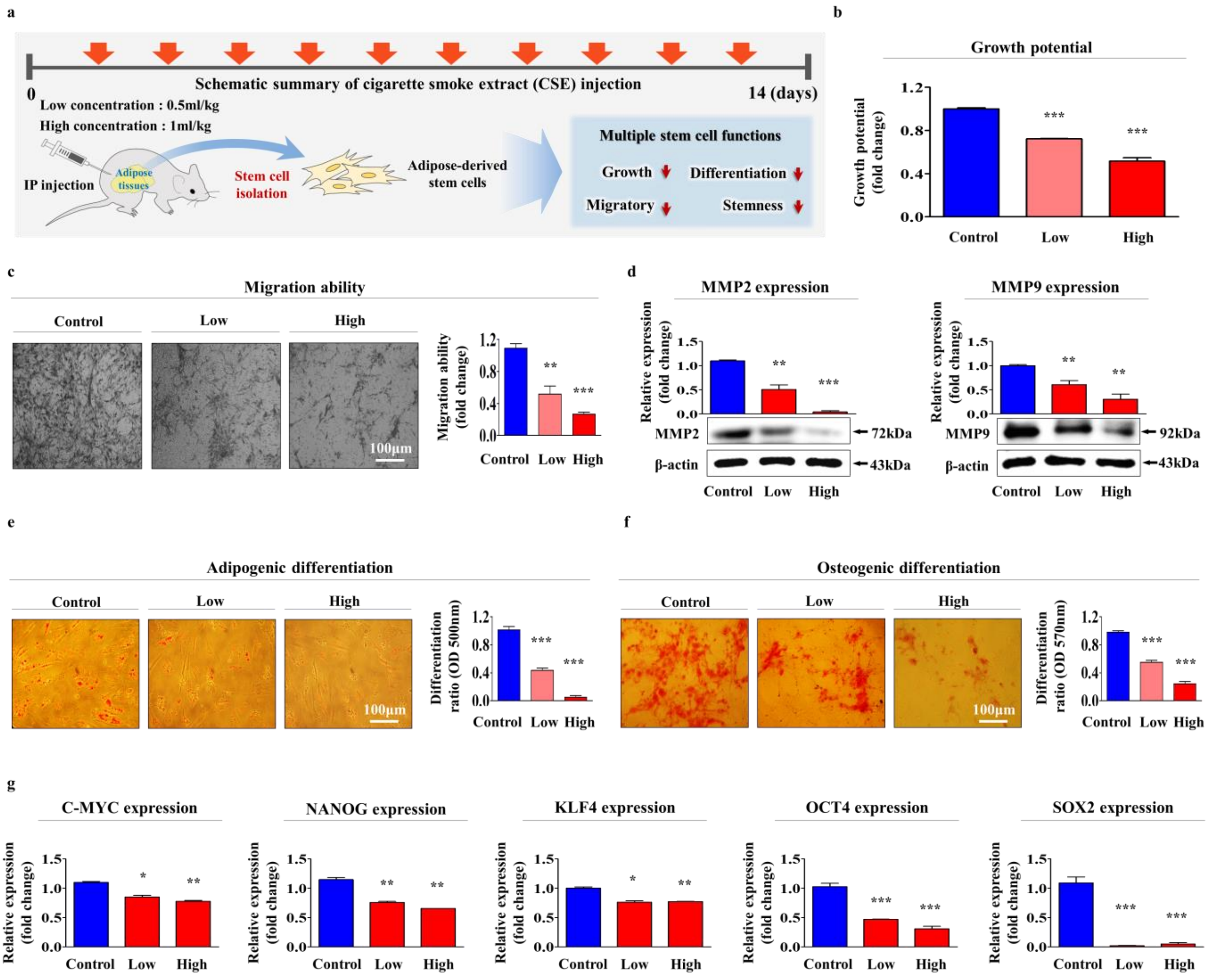

Supplementary fig. 7 Cigarette smoke exposure markedly suppressed various tissue regeneration-associated functions of adipose tissue-derived stem cells *in vivo*. Schematic of the *in vivo* experimental procedure described in Materials and Methods **(a)**. Mice were intraperitoneally administrated a low (0.5 mg/kg) or high (1 mg/kg) dose of cigarette smoke extract or vehicle (medium) 10 times. Adipose tissue-derived stem cells were then isolated from mouse adipose tissues, and the inhibitory effects of cigarette smoke exposure on cell viability were assessed using an MTT assay **(b)**. The changes in stem cell migratory abilities were determined using a transwell assay **(c)** and by western blotting using MMP-2 and -9 antibodies **(d)**. The inhibitory effects of cigarette smoke extract

on the differentiation of mouse adipose tissue-derived stem cells into adipocytes **(e)** and osteoblast **(f)** *in vivo* were analyzed by oil red O and alizarin red staining, respectively. Relative quantifications of calcium mineral contents and lipid droplet formation within differentiated cells were evaluated by measuring absorbance at 500 and 570 nm, respectively. The inhibitory effects of cigarette smoke exposure on the expressions of various pluripotency-associated genes, C-MYC, KLF4, NANOG, OCT4, and SOX2, were determined by real-time PCR **(g)**. β-actin was used as the internal control, and PPIA as the housekeeping gene for real-time PCR analysis. All experiments were performed in triplicate. Data are presented as means ± SDs. *, $p < 0.05$; **, $p < 0.005$; and ***, $p < 0.001$ (by the two-sample t-test).

Supplementary Fig. 8

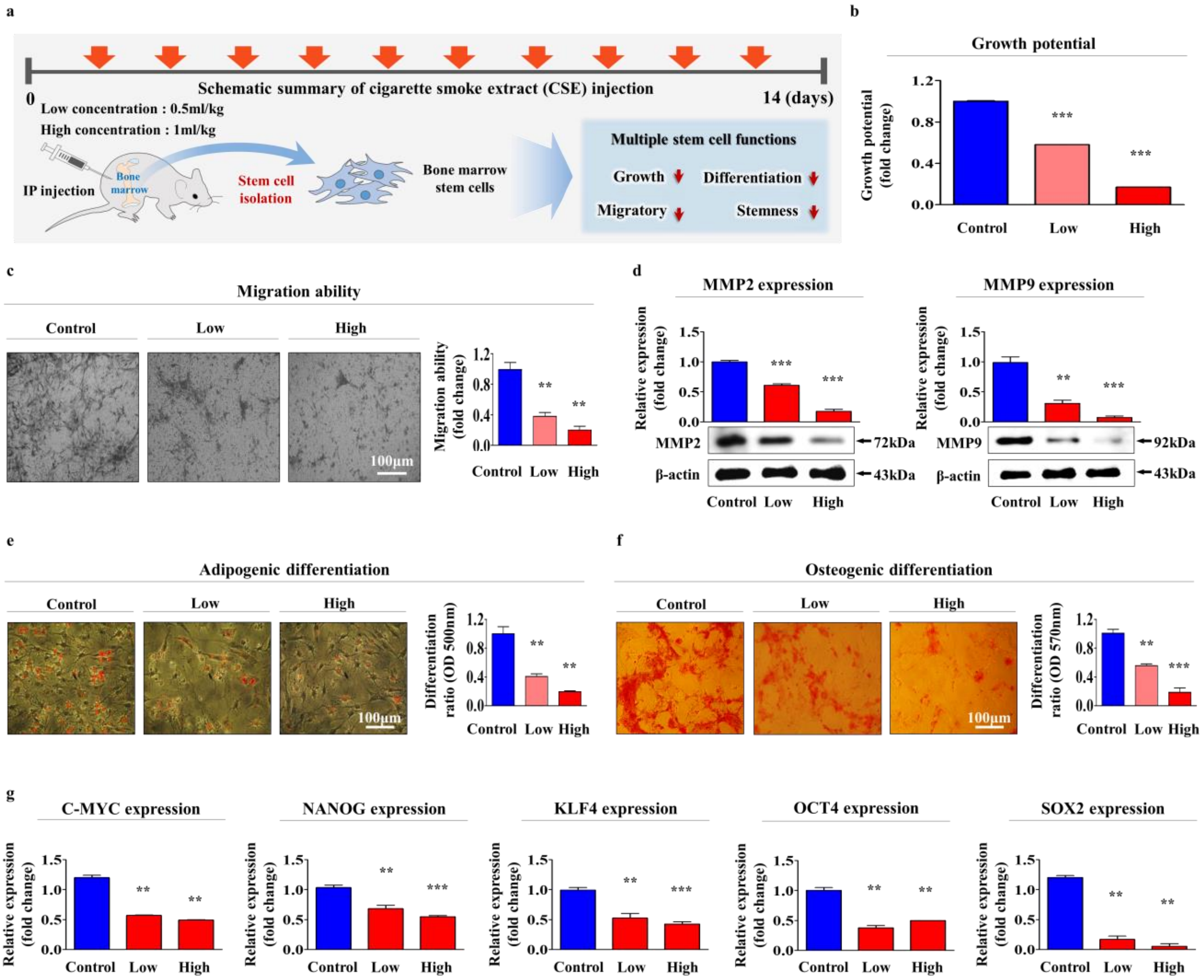

Supplementary fig. 8 Cigarette smoke exposure markedly suppressed various tissue regeneration-associated functions of bone marrow-derived stem cells *in vivo*. Schematic of the *in vivo* experimental procedure described in Materials and Methods **(a)**. Mice were intraperitoneally administrated low (0.5 mg/kg) or high (1 mg/kg) dose of cigarette smoke extract 10 times or vehicle (medium). Bone marrow-derived stem cells were isolated from bone marrow, and the inhibitory effects of cigarette smoke exposure on cell viability were assessed using an MTT assay **(b)**. The changes in stem cell migratory abilities were analyzed using a transwell assay **(c)** and western blotting was performed using MMP-2 and -9 antibodies **(d)**. The inhibitory effects of cigarette smoke exposure on the differentiations of mouse bone marrow-derived stem cells into adipocytes **(e)** or

osteoblasts **(f)** *in vivo* were assessed by oil red O and alizarin red staining, respectively. Relative quantifications of calcium mineral contents and lipid droplet formation within differentiated cells were performed by measuring absorbances at 500 and 570 nm, respectively. The inhibitory effects of cigarette smoke exposure on the expressions of various pluripotency-associated genes, C-MYC, KLF4, NANOG, OCT4, and SOX2, were analyzed by real-time PCR **(g)**. β-actin was used as the internal control, and PPIA as the housekeeping gene for real-time PCR analysis. All experiments were performed in triplicate. Data are presented as means ± SDs. *, $p < 0.05$; **, $p < 0.005$; and ***, $p < 0.001$ (two-sample t-test).
